# Comparative genomics reveals a novel genetic organization of the *sad* cluster in the sulfonamide-degrader ‘*Candidatus* Leucobacter sulfamidivorax’ strain GP

**DOI:** 10.1186/s12864-019-6206-z

**Published:** 2019-11-21

**Authors:** Ana C. Reis, Boris A. Kolvenbach, Mohamed Chami, Luís Gales, Conceição Egas, Philippe F.-X. Corvini, Olga C. Nunes

**Affiliations:** 1 Laboratory for Process Engineering, Environment, Biotechnology and Energy, Faculty of Engineering - LEPABE, Department of Chemical Engineering, University of Porto, Rua Dr. Roberto Frias s/n, 4200-465 Porto, Portugal; 2 Institute for Ecopreneurship, School of Life Sciences, University of Applied Sciences Northwestern Switzerland, Gruendenstrasse 40, 4132 Muttenz, Switzerland; 3 BioEM lab, C-Cina, Biozentrum, University of Basel, Mattenstrasse 26, CH-4058 Basel, Switzerland; 4 Instituto de Investigação e Inovação em Saúde - i3S, Rua Alfredo Allen 208, 4200-135 Porto, Portugal; 5 Instituto de Biologia Molecular e Celular - IBMC, Rua Alfredo Allen 208, 4200-135 Porto, Portugal; 6 Instituto de Ciências Biomédicas Abel Salazar - ICBAS, Rua de Jorge Viterbo Ferreira 228, 4050-313 Porto, Portugal; 7 Next Generation Sequencing Unit, Biocant, BiocantPark, Núcleo 04, Lote 8, 3060-197 Cantanhede, Portugal; 8 Center for Neuroscience and Cell Biology, University of Coimbra, Faculty of Medicine, Rua Larga, Pólo I, 3004-504 Coimbra, Portugal

**Keywords:** Sulfonamides, Bacterial consortium, Phylogenetic analysis, Metagenome-assembled genome, Cryo-transmission electron microscopy

## Abstract

**Background:**

Microbial communities recurrently establish metabolic associations resulting in increased fitness and ability to perform complex tasks, such as xenobiotic degradation. In a previous study, we have described a sulfonamide-degrading consortium consisting of a novel low-abundant actinobacterium, named strain GP, and *Achromobacter denitrificans* PR1. However, we found that strain GP was unable to grow independently and could not be further purified.

**Results:**

Previous studies suggested that strain GP might represent a new putative species within the *Leucobacter* genus (16S rRNA gene similarity < 97%). In this study, we found that average nucleotide identity (ANI) with other *Leucobacter* spp. ranged between 76.8 and 82.1%, further corroborating the affiliation of strain GP to a new provisional species. The average amino acid identity (AAI) and percentage of conserved genes (POCP) values were near the lower edge of the genus delimitation thresholds (65 and 55%, respectively). Phylogenetic analysis of core genes between strain GP and *Leucobacter* spp. corroborated these findings. Comparative genomic analysis indicates that strain GP may have lost genes related to tetrapyrrole biosynthesis and thiol transporters, both crucial for the correct assembly of cytochromes and aerobic growth. However, supplying exogenous heme and catalase was insufficient to abolish the dependent phenotype. The actinobacterium harbors at least two copies of a novel genetic element containing a sulfonamide monooxygenase (*sad*A) flanked by a single IS1380 family transposase. Additionally, two homologs of *sad*B (4-aminophenol monooxygenase) were identified in the metagenome-assembled draft genome of strain GP, but these were not located in the vicinity of *sad*A nor of mobile or integrative elements.

**Conclusions:**

Comparative genomics of the genus *Leucobacter* suggested the absence of some genes encoding for important metabolic traits in strain GP. Nevertheless, although media and culture conditions were tailored to supply its potential metabolic needs, these conditions were insufficient to isolate the PR1-dependent actinobacterium further. This study gives important insights regarding strain GP metabolism; however, gene expression and functional studies are necessary to characterize and further isolate strain GP. Based on our data, we propose to classify strain GP in a provisional new species within the genus *Leucobacter*, ‘*Candidatus* Leucobacter sulfamidivorax‘.

## Background

Microbial communities are known to establish sophisticated metabolic interactions in order to achieve complex and energy-expensive tasks [1–5]. These syntrophic relationships are frequently studied in bacterial pathogens and symbiotic bacteria, where the interaction with the host often drives progressive adaptation, mutation, and subsequently, gene loss. These phenomena may render the bacteria “unculturable” or difficult to grow under standard laboratory conditions [6–11]. On the contrary, the phenomena underlying metabolic cooperation and competition within environmental communities are often more complex, and their implications for microbial ecology are still poorly understood [5, 11]. These communities recurrently exchange metabolites or co-factors and are often associated with xenobiotic-degraders thriving in polluted environments [5, 11–15]. This syntrophy has been previously observed in terephthalate-degrading communities [1, 2], in anammox-associated communities [3, 4, 16], in the dichloromethane-degrader ‘*Candidatus* Dichloromethanomonas elyunquensis’ [17], and in members of the candidate phylum ‘*Candidatus* Latescibacteria’, that thrives in hydrocarbon-impacted environments [18, 19]. However, to date, no representatives of these groups could be isolated as pure cultures, and their metabolic needs are difficult to assess. Terephthalate-degraders, for instance, thrive in an intricate network formed between H_2_-producing syntrophs and methanogenic archaea, with numerous other secondary interactions essential for the stability of the consortium [1, 2]. Anammox bacteria were shown to form stable biofilm communities with ammonia-oxidizing bacteria (AOB), that appear to be essential to protect the sensitive anammox species from atmospheric O_2_ [3, 4, 20, 21]. The evolution of these communities is driven by selective pressure and stress and may result in complex syntrophic relationships that may lead to niche-specialization and dependency on other members of the community. In order to characterize the members of these communities, cell-sorting and metagenomics approaches are being used to circumvent the need for cultivation [15]. Furthermore, these studies are frequently complemented with comparative genomics which has emerged as a valuable tool to determine the evolution and functional prediction between even distantly related bacteria [14, 22, 23]. The cultivation of several members of the ubiquitous SAR11 aquatic bacteria, with no closely related culturable relatives, has been made possible by in silico metabolic studies and next-generation sequencing approaches [24]. Furthermore, the evolution of this abundant group of *Alphaproteobacteria* and their ecological importance has been further elucidated using comparative genomic approaches [25]. In a previous study, we have described a microbial consortium between *Achromobacter denitrificans* strain PR1 and strain GP that depends on strain PR1’s presence for growth [26]. Strain GP showed the highest pairwise similarity of its 16S rRNA gene sequence to members of the genus *Leucobacter*. Independently of the tested culture media, cofactors and culture conditions no pure cultures were obtained for strain GP [26]. To characterize strain GP, we have sequenced the two-member consortium and reconstructed its draft genome. Also, we have performed comparative genomic studies in order to understand its phylogenetic relationship with other members of the *Leucobacter* genus and propose the hypothesis that may allow us to understand why this strain has eluded isolation in previous studies.

## Results and discussion

### Morphological and physiological characterization of the consortium

**Fig. 1:**
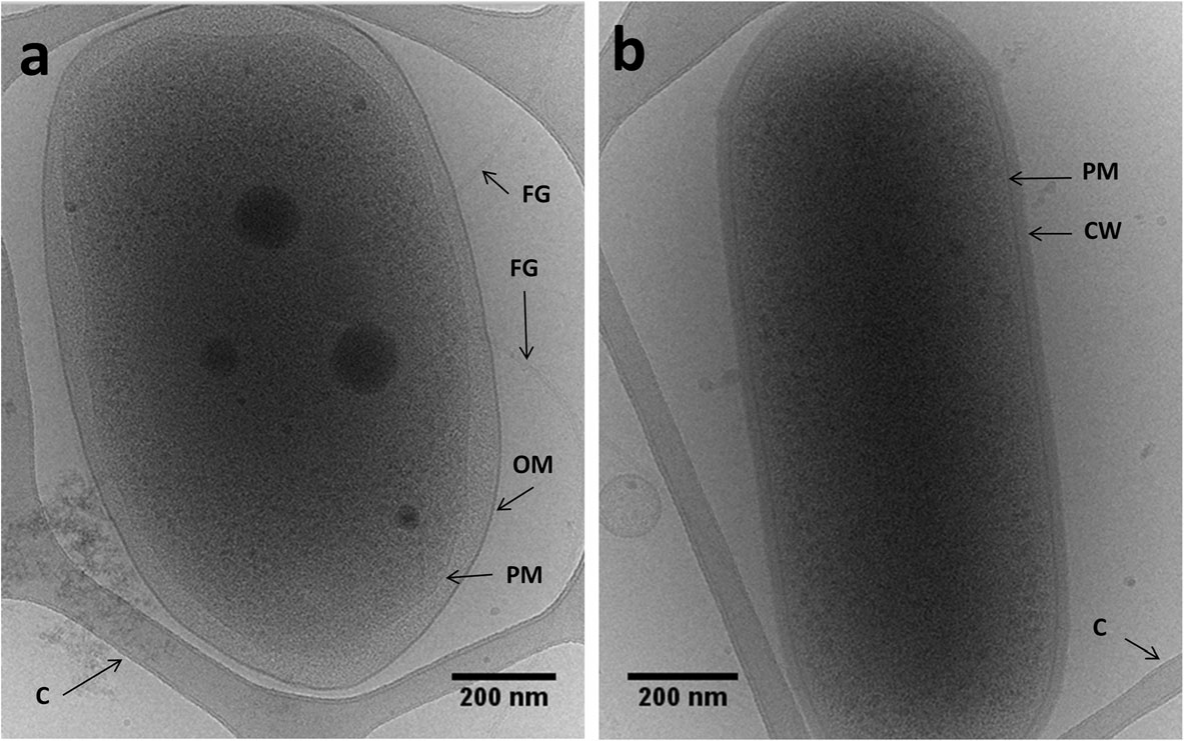
Electron micrographs of frozen hydrated *Achromobacter denitrificans* strain PR1 (**a**) and strain GP (**b**). PM – Plasma membrane; OM – Outer membrane; FG – Flagellum; CW – Cell wall; C – Carbon support grid

**Fig. 2:**
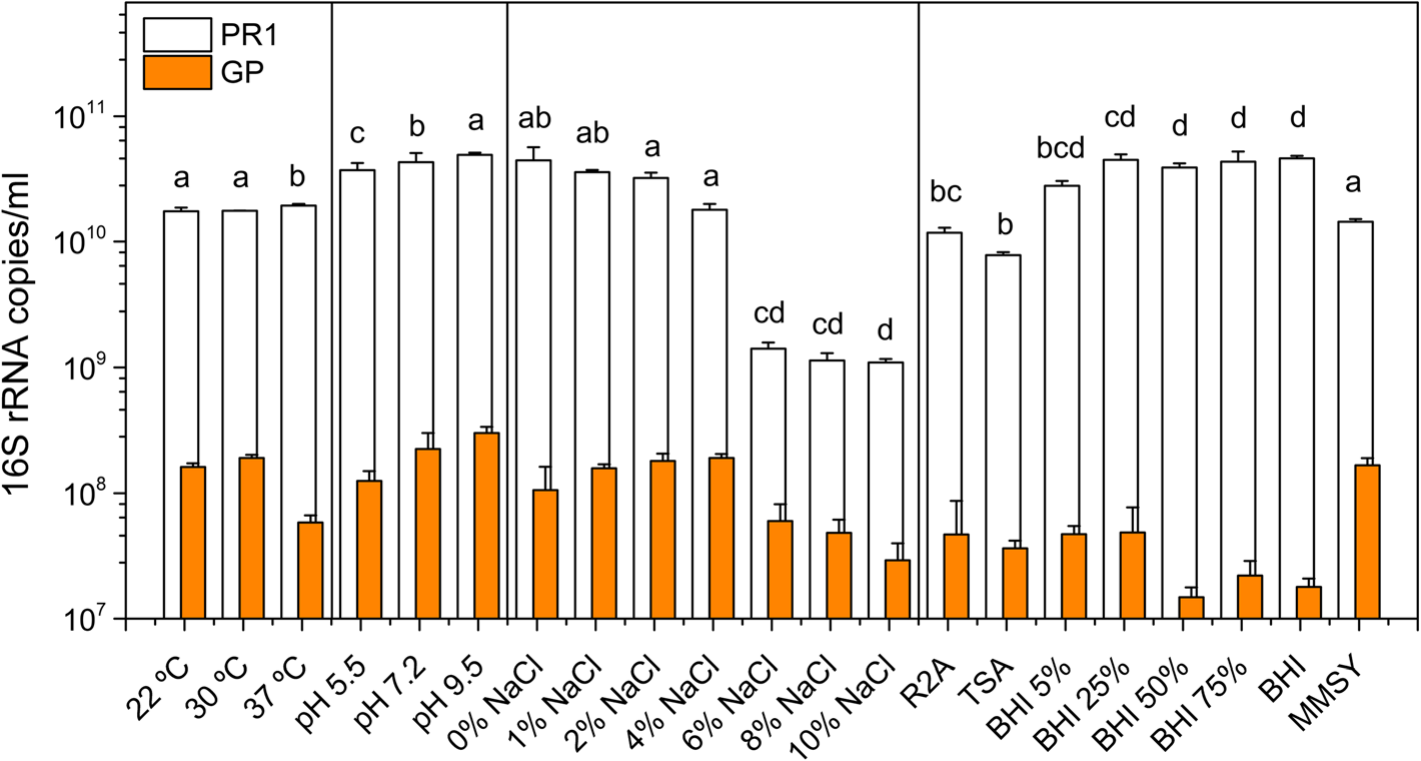
Abundance of strain PR1 and strain GP after 15 h incubation at different pH, salinity (in DLB), temperatures (in MMSY) and media (R2A, TSA, BHI and MMSY). The values for copies of the 16S rRNA gene per ml are plotted in logarithmic scale. Values are the mean values of triplicates and the error bars represent the standard deviation. Significant differences in strain GP abundance are indicated by a, b, c and d (from higher to lower values of the mean) as determined by two-way ANOVA (pH, temperature and salinity) or one-way ANOVA (PR1/GP ratio in R2A, TSA, BHI and MMSY) and the Tukey test at *p* < 0.05 within each tested condition [33]

The microbial consortium between strain *A. denitrificans* and the low-abundant strain GP was visualized by Cryo-TEM during mid-stationary phase (Fig. 1a and b), as well as by FISH (see Additional file 1 Figure S1). As expected, strain PR1 showed the typical morphology of Gram-negative rods with an average cell size of 801.3 ± 40.2 nm (width), 1332 ± 98.7 nm (length) and 38.2 ± 6.5 nm (periplasmic space) (Fig. 1a). Moreover, peritrichous flagella were observed by negative stain electron microscopy (FG, see Additional file 1 Figure S2). Although flagella have not been previously reported for the type strain of *A. denitrificans*, their presence has been repeatedly observed in other strains from this species [27] and other species of the *Achromobacter* genus [28, 29]. Conversely, strain GP displayed the typical morphology of Gram-positive rods. Its cells showed an average size of 506.6 ± 30.1 nm (width) and 1341.0 ± 29.7 nm (length) (Fig. 1b), and the rigid cell wall of this organism had an average thickness of 20.6 ± 2.2 nm. No flagella were observed for this bacterium, suggesting that it is non-motile, like previously reported for other members of the *Leucobacter* genus [30, 31]. The two members of the consortium revealed significant differences regarding their respective tolerances toward temperature, pH and salinity (Fig. 2). While the abundance of strain PR1 was constant when incubating at 22, 30 and 37 °C, respectively, strain GP abundance was significantly reduced at 37 °C (*p* < 0.05) when compared to the other tested temperatures. Strain GP also showed a lower abundance when incubated at pH 5.5, in comparison to cultures incubated in media at neutral (pH 7.2) and basic (pH 9.5) pH values (Fig. 2). As it is typically observed for members of the *Achromobacter* genus [32], NaCl concentrations up to 4% (w/v) did not influence the abundance of strain PR1; however, its abundance was significantly reduced above this value (Fig. 2). Although the absolute amount of strain GP 16S rRNA copy numbers also decreased above 4% NaCl (w/v), the relative abundance of this strain in the consortium was significantly higher (ranging from 0.24% at 0% NaCl, to a maximum of 4.26% at 8% NaCl). Interestingly, the abundance of strain GP was significantly lower in complex media (Tryptic Soy Broth, TSA; Brain-Heart Infusion, BHI; and Reasoner’s 2A medium, R2A) than in mineral media with succinate and trace amounts of yeast extract (MMSY, Fig. 2). These results suggest that strain GP is possibly oligotrophic, unlike previously described for members of the *Leucobacter* genus, which thrive in complex media, such as BHI enriched with peptone and yeast extract, as observed for *L. luti* RF6^T^ [30].

### Analysis of the metagenome-assembled genome of strain GP

The analysis of the metagenomic contigs with SSU finder (rRNA small subunit) from CheckM [34] revealed the presence of only two phylogenetic distinct organisms: one identified as *A. denitrificans* PR1 and the other as strain GP. The reconstruction of strain GP’s genome from whole-consortium sequencing generated a metagenome-assembled genome (MAG) consisting of 11 contigs, with 3.84 Mb, 3621 coding sequences (CDS), 69.68% in G + C and a total mapped coverage of 61x (Table 1). In spite of an enrichment step with 2-phenylethanol, only 18.5% of the total of reads obtained with Oxford Nanopore (ONT) and Illumina technologies were mapped to strain’s GP MAG, while the remaining reads mapped to the complete genome of *A. denitrificans* PR1 (148x coverage in the consortium, see Additional file 2 Table S1), previously determined [35]. The MAG of strain GP encoded a complete rRNA operon and harbored two copies of the 5S and one copy of the 16S and 23S rRNA subunits, respectively. Moreover, analysis with tRNAscan-SE [36] identified 44 tRNA encoding for all 20 amino acids. CheckM [34] analysis showed high completeness and low contamination values for this assembly, as only 7 marker genes were not detected in the draft genome and 3 markers had 2 copies in the assembly (95.9% completeness and 0.6% contamination, respectively, see Additional file 2 Table S2). Therefore, according to Bowers et al. [37], these findings indicate that this methodology allowed the reconstruction of a high-quality MAG for strain GP (Table 1).

**Table 1:** List of named species of the *Leucobacter* genus used in the phylogenetic and comparative studies. Assembly quality was calculated using QUAST [49] with a minimum contig size set to 200 bp. Completeness and contamination were computed with CheckM [34]. 16S rRNA pairwise similarity was computed with the global alignment tool in the EzBioCloud web server [126]

Strain	16S rRNA pairwise similarity to GP (%)	Genome*/ assembly accession no.	Completeness (%)	Contamination (%)	Number contigs/ scaffolds	Genome size (Mb)	Contig N50	G + C content (%)	Number of CDS	Reference
*Candidatus* Leucobacter sulfamidivorax’ GP	–	QZLF00000000	95.91	0.58	11	3.84	956,104	69.68	3621	This study
*L. aridicollis* L-9^T^	96.23	QYAE00000000	99.27	0.58	8	3.56	888,847	67.3	3212	This study
*L. celer* subsp. *astrifaciens* CBX151^T^	95.87	GCA_001273835.1	98.83	0.00	235	4.14	349,813	69.1	3661	[122]
*L. chironomi* DSM 19883^T^	95.43	GCA_000421845.1	100.00	0.88	27	2.96	268,438	69.9	2662	[64]
*L. chromiireducens* subsp. *chromiireducens* L-1^T^	96.23	QYAD00000000	100.00	0.58	6	3.22	623,960	67.0	2843	This study
*L. chromiireducens* subsp. *solipictus* TAN 31504^T^	96.15	QYAC00000000	99.42	2.05	11	3.54	451,461	68.9	3096	This study
*L. chromiiresistens* J31^T^	96.23	GCA_000231305.1	100.00	0.00	2	3.21	2,823,343	70.3	2895	[31]
*L. chromiiresistens* NS354	96.37	GCA_001477055.1	95.03	0.00	194	2.79	35,220	70.8	2423	[142]
*L. komagatae* DSM 8803^T^	96.79	GCA_006716085.1	98.54	1.75	2	3.75	3,292,530	66.6	3253	Unpublished
*L. luti* RF6^T^	96.81	QYAG00000000	100.00	0.58	5	3.62	1,858,864	69.4	3088	This study
*L. massiliensis* 122RC15^T^	96.52	GCA_002982315.1	100.00	1.75	24	3.14	232,656	71.0	2789	[143]
*L. musarum* subsp. *japonicus* CBX130^T^	95.87	GCA_001273855.1	99.56	0.58	144	3.59	248,155	66.8	3311	[144]
*L. musarum* subsp. *musarum* CBX152^T^	95.87	GCA_001273845.1	99.85	0.58	125	3.44	200,785	66.8	3147	[144]
*L. salsicius* M1-8^T^	95.94	GCA_000350525.1	99.42	0.00	28	3.18	197,637	64.5	2741	[65]
*L. triazinivorans* JW-1^T^	96.4	GCA_004208635.1	100.00	0.88	1	3.48	–	69.37	2978	[145]
*L. zeae* CC-MF41^T^	95.88	QYAB00000000	99.85	0.58	6	3.47	2,226,772	70.6	3042	This study

### Analysis of mobile and conjugative elements

The identification of potential plasmids and other mobilizable elements in the genome of strain GP was performed in silico by measuring differences in coverage and G + C content between the contigs of the draft assembly. Compared to the average values for all contigs, at least three contigs (5, 7 and 9) showed a significantly higher coverage, and lower G + C content (see Additional file 2 Table S1). The differential coverage among contigs was observed consistently with both Illumina and ONT libraries, which were prepared from different biological replicates of the consortium. Therefore, these differences are unlikely to arise from library preparation and sequencing bias. The differences encountered suggest that these contigs may represent potential plasmids with an average copy number per cell of approximately 2–3 (contigs 5 and 9) and 9 (contig 7), respectively. Furthermore, conserved domain search and CONJscan revealed the presence of several elements linked to plasmid replication, stability, partitioning, conjugation, and mobility (Table 2). Out of these three contigs, only contig 9 (11.8 kb) was marked as circular by Circlator [38]; however, it had no relevant hits to other plasmids available in the National Center for Biotechnology Information (NCBI) database. Contrarily, contig 7 (22.5 kb) featured residual homology to a new plasmid found in *Cnuibacter physcomitrellae* XA^T^ (accession number CP020716.1, 4285 bp alignment with 99% identity to this plasmid), and the plasmid pKpn-35963cz from *Klebsiella pneumoniae* Kpn-35963cz (accession number MG252894.1, 2030 bp alignment with 99% identity to this plasmid). The respective homologous regions contained genes encoding for transposases and mercury resistance. Both contigs 7 and 9 carry a gene encoding for a putative relaxase (locus tag: D3X82_18105, D3X82_18250, respectively) with a TrwC family domain (accession no. pfam08751; E-value: 3.7e-28 and 7.6e-25, respectively), commonly observed in proteins from the MOB_F_ (mobility) family (e.g., TraA from *Arthrobacter* sp. Chr15, accession no. ABR67091.1 [39]). This classification was further confirmed by CONJscan [40–42], which found that both D3X82_18105 (contig 7) and D3X82_18250 (contig 9) possess a highly conserved MOB_F_ domain (E-values of 5.3e-105 and 4.1e-106, respectively). Additional mobility elements were only found in contig 7. This contig was found to harbor a putative plasmid replication protein (locus tag: D3X82_18090; Family: RepA_C; accession no: pfam04796; E-value: 9.0e-07). In this way, according to Guglielmini et al. [42] and Smillie et al. [43], the presence of a MOB element in contigs 7 and 9 suggests these putative elements are mobilizable but non-conjugative. Contig 5, with 74 kb, was found to contain various integrative and conjugative elements (Table 2) [44]. Besides, this contig contained all antimicrobial resistance genes found in the genome of strain GP (*sul*1, *tet*(33), *aad*A1, *qac*E), as well as two copies of the *sad*A gene encoding for the previously described sulfonamide monooxygenase [26]. Table 2 Homology searches for contig 5 against the NCBI database [45] revealed residual homology to *Enterobacter cloacae* strain EclC2185’s genomic island (accession number MH545561.1, 5187 bp alignment with 99% identity to the genomic island of this strain) containing a class I integron with multi-drug resistance genes (*aad*A1, *sul*1, and *qac*E). Other significant alignments included regions conferring mercury resistance (*Cnuibacter physcomitrellae* XA^T^ plasmid, accession number CP020716.1, 5928 bp alignment with 99% identity to this plasmid) and intergenic regions of the new plasmid pOAD2 from *Flavobacterium* sp. KI723TI (accession number D26094.1, 14,820 bp alignment with 94% to this plasmid). According to conserved domain search and CONJscan analyses, two putative MOB elements were found in contig 5: (i) D3X82_17470, a relaxase from the MOB_F_ family with a TwrC conserved domain (CONJscan domain search: E-value 1.3e-85); (ii) D3X82_17405, a relaxase from the MOB_P1_ family (CONJscan domain search: E-value 4.2e-40). Other essential mobilizable elements detected include a type IV coupling protein (T4CP, locus tag D3X82_17390) with a conserved VirD4 domain (CONJscan domain search: E-value 5.7e-40) and a type IV secretion protein (T4SS, locus tag D3X82_17385) with a VirB4 domain (CONJscan domain search: E-value 1.4e-25). According to Smillie et al. [43], these three elements (T4SS, T4CP and relaxases), represented in four locus tags in strain GP, are at the core of plasmid conjugation, however, no other known accessory proteins were detected in our analysis, presumably due to incomplete assembly and/or low identity to previously characterized proteins from the mating-pair formation (MPF) system. In this way, no complete type IV secretion systems were detected in contig 5 suggesting this element may be mobile but possibly not conjugative.

**Table 2:** Genes and corresponding conserved domains linked to integrative, conjugative and resistance elements found in contigs 5, 7 and 9 from the draft assembly of strain GP. Families and E-values in bold indicate the best hits obtained with CONJscan [146]. n.a., not applicable

System	Contig	Locus tag	Description	Family	Accession	E-value
Replication, stabilization and partitioning	5	D3X82_17420	Toxin protein from a toxin/antitoxin system	Zeta_toxin	pfam06414	1.6e-19
	5	D3X82_17550	Plasmid partition protein A	BcsQ partition_RepA	COG1192 TIGR03453	2.2e-43 5.7e-16
	5	D3X82_17555	Single-stranded DNA-binding protein	SSB_OBF	cd04496	8.5e-12
	7	D3X82_18090	Plasmid replication protein	RepA_C	pfam04796	9.0e-07
Mobilization and conjugative elements	5	D3X82_17410	Plasmid mobilization relaxosome protein MobC	MobC	pfam05713	5.7e-05
	5	D3X82_17470	Conjugal transfer protein	TrwC **MOB**_F_	pfam08751 n.a.	9.0e-97 1.3e-85
	5	D3X82_17385	Type IV secretion protein (T4SS)	**VirB4**	n.a.	1.40e-25
	5	D3X82_17390	Coupling protein (T4CP)	VirD4 **T4CP2**	COG3505 n.a.	4.2e-16 5.7e-40
	5	D3X82_17405	Relaxase	Relaxase **MOB**_P1_	pfam03432 n.a.	9.8e-10 4.20e-40
	7	D3X82_18105	Conjugal transfer protein	TrwC **MOB**_F_	pfam08751 n.a.	3.7e-28 5.3e-105
	9	D3X82_18250	Conjugal transfer protein	TrwC **MOB**_F_	pfam08751 n.a.	7.6e-25 4.1e-106
Multi-drug and heavy metal resistance	5	D3X82_17365 D3X82_17695	Sulfonamide monooxygenase SadA	NcnH CaiA Acyl-CoA_dh_2	cd01159 COG1960 pfam08028	1.52e-84 1.93e-30 5.8e-20
	5	D3X82_17485	Tet(A)/Tet(B)/Tet(C) family tetracycline efflux MFS transporter	MFS_TetA	cd17388	2.4e-145
	5	D3X82_17505	ANT(3″)-Ia family aminoglycoside nucleotidyltransferase AadA1	PRK13746 DUF4111	PRK13746 pfam13427	0e+ 00 1.31e-41
	5	D3X82_17510	Quaternary ammonium compound efflux SMR transporter QacE delta 1	Multi_Drug_Res	pfam00893	1.75e-28
	5	D3X82_17515	Sulfonamide-resistant dihydropteroate synthase Sul1	Pterin_bind	pfam00809	1.8e-87
	5	D3X82_17685	Mercury(II) reductase	MerA	TIGR02053	1.2e-171

### Phylogenetic analysis

**Fig. 3:**
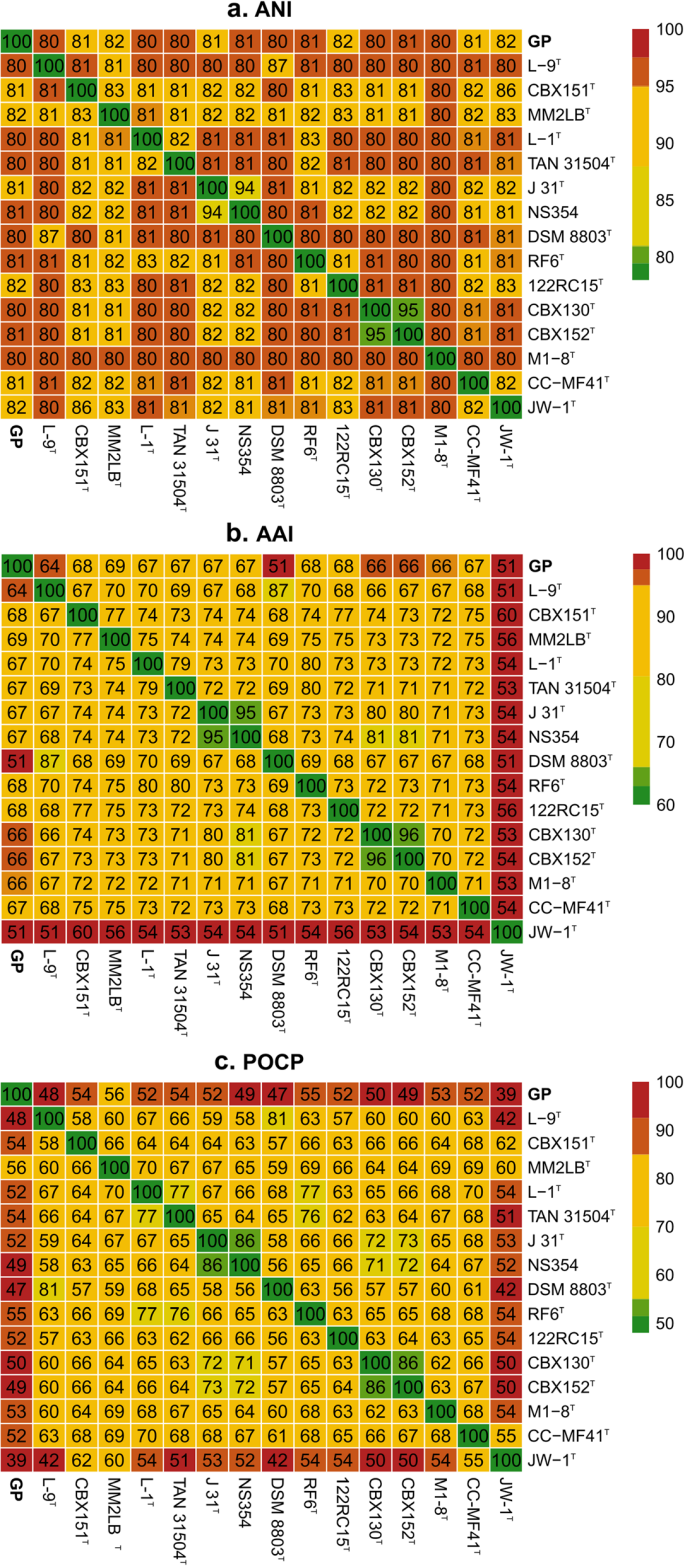
ANI (**a**), AAI (**b**) and POCP (**c**) heatmaps comparing values between strain GP and validly named species of the *Leucobacter* genus at the time of analysis

**Fig. 4:**
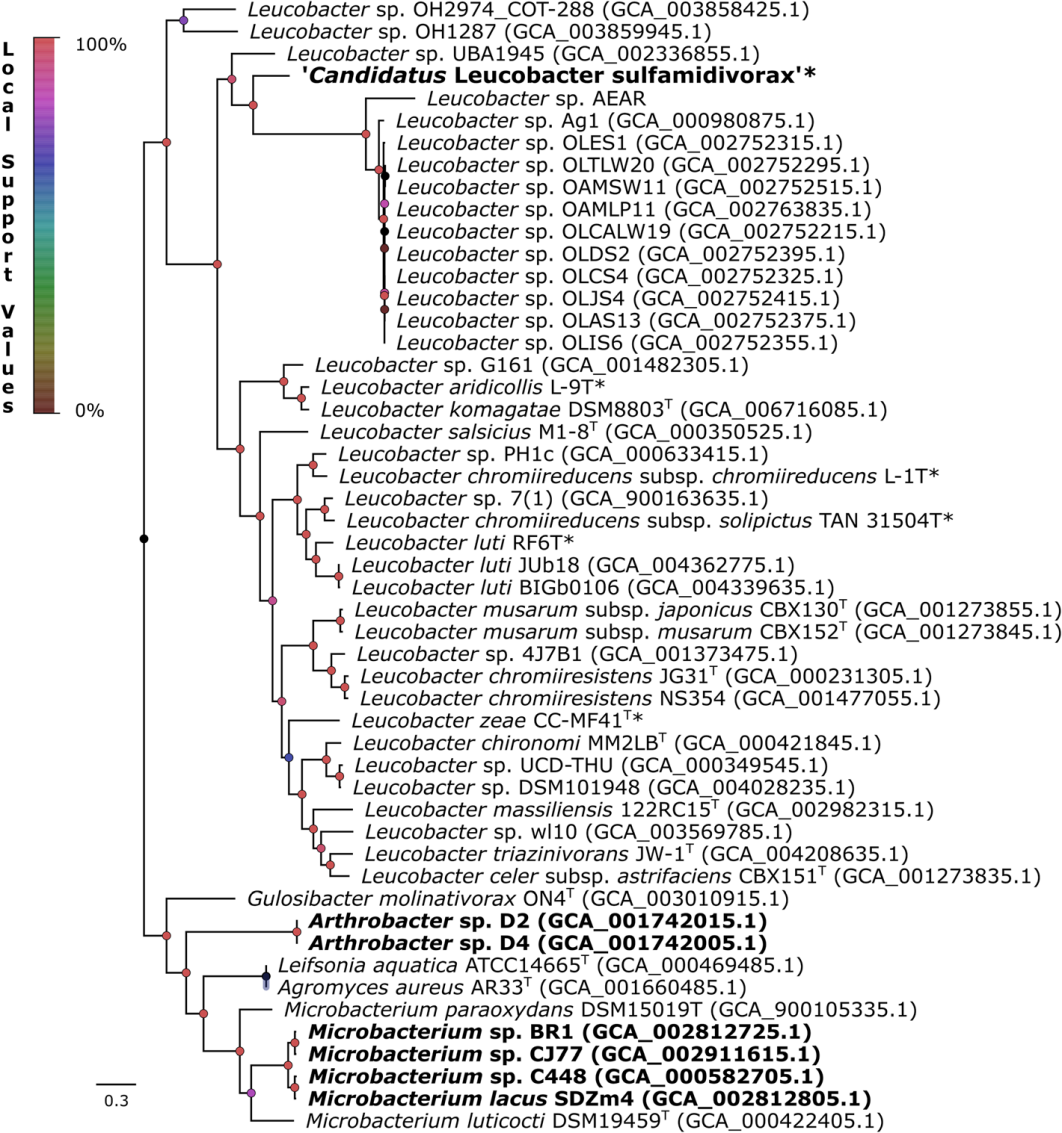
Phylogenomic relationships between the *Leucobacter* genus and strain GP inferred from concatenated amino acid alignments of 400 universal proteins obtained with PhyloPhlAn [52]. Representative members of genera *Microbacterium*, *Leifsonia*, *Gulosibacter*, *Agromyces* a n d *Arthrobacter* were included as outgroup. *Leucobacter* spp. strains sequenced in this study are marked with an asterisk, and sulfonamide degraders are shown in bold. Node labels indicate local support values obtained with FastTree using the Shimodaira-Hasegawa test [53]. The scale bar represents the number of expected substitutions per site. The tree was rooted at the outgroup node and visualized with FigTree [125]

As reported previously, strain GP shares the highest 16S rRNA gene sequence similarity with members of the genus *Leucobacter,* 94.6–96.9% (see Additional file 2 Table S3), below the 98.7% threshold currently used to define a new species [26, 46] and close to the 97% threshold used to define a new genus [47]. The phylogenetic analysis inferred from the alignment of the near-complete 16S rRNA gene between all fully sequenced *Leucobacter* spp. showed that strain GP indeed clusters with *Leucobacter* spp. (see Additional file 1 Figure S3). Nevertheless, the ANI values between strain GP and the type strains of the validly named species of this genus ranged between 80.0 and 82.1% (Fig. 3a, Additional file 2 Table S3), well below the general species delimitation thresholds (94–96%) [48, 49], indicating that strain GP could not be affiliated to any of these species. Average amino acid identity (AAI) comparisons between this strain and the type strains of the validly named species of this genus ranged between 64.2 and 69.1% (Fig. 3b, Additional file 2 Table S3). These values are near the lower edge of the typical genus delimitation boundaries (approximately 65%) [49], and the specific interspecies boundaries found between the analyzed type strains of *Leucobacter* spp. (51.0–87.3%). This result was further supported by the percentage of conserved genes (POCP) [50]. POCP values ranged between 46.7 and 56.5% (Fig. 3c, Additional file 2 Table S3), which is also on the lower edge of the interspecies boundaries found for this genus (42.0–81.3%) and the value suggested by Qin et al. [50] for new genus delimitation (55%). The G + C content of strain GP was of 69.7% (Table 1), which, according to previous studies [51] is within the expected G + C interval (10%) for organisms of the same genus. In fact, for the type strains of all validly named species of the *Leucobacter* genus, G + C content ranged between 64.5 and 71.0% (Table 1). Moreover, the phylogenetic analysis of 400 conserved proteins of *Leucobacter* spp. using the PhyloPhlAn pipeline [52] revealed that although strain GP appears to share a common origin with the other isolates of *Leucobacter* spp. (Fig. 4), it also does not cluster with any of the analyzed strains.

### Core and softcore genome of *Leucobacter* spp.

Orthologs gene cluster analysis with GET_HOMOLOGUES [54] revealed that *Leucobacter* spp. and strain GP core and softcore genome contain 456 and 885 orthologs gene clusters, respectively (see Additional file 1 Figure S4). However, only a fraction of these (approximately 50%) could be functionally annotated with eggNOG-Mapper and BlastKOALA [55, 56]. This analysis revealed that most of these clusters are related to central metabolic pathways [57], including nucleotide and amino acid metabolism (118 clusters), and carbohydrate and lipid metabolism (16 clusters) (see Additional file 2 Table S4), respectively. Furthermore, these strains lack orthologs linked to antimicrobial resistance, quorum sensing, and biofilm formation, suggesting that they form a diverse and versatile genus with specific adaptations to different environments (see Additional file 2 Table S4). Only a few of the fully sequenced *Leucobacter* spp. analyzed are free-living organisms isolated from wastewater or soil. These free-living strains did not form a clade. The majority of the strains form facultative symbiotic associations with arthropods, nematodes, and plants (see Additional file 2 Table S3). While *Leucobacter* sp. AEAR [58], whose genome has been directly reconstructed from whole genome sequences of the nematodes *Caenorhabditis angaria* and *Caenorhabditis remanei*, could not be isolated, all *Leucobacter* spp. symbionts were able to grow independently from their hosts. Nevertheless, the analysis of strain’s AEAR genome revealed that it should be able to grow independently as all essential pathways seem to be present in its draft genome [58]. This observation is further supported by the analysis of the genome of this strain (see Additional file 2 Table S3). Unlike obligate symbionts, which often undergo extreme genome reduction [59–62], strain AEAR possesses a genome with similar size (3.54 Mb) and genetic density when compared to its closest relatives (Fig. 4). Moreover, strain AEAR forms a monophyletic clade with *Leucobacter* sp. Ag1 (accession no. GCA_000980875.1) and other 9 strains, which are all facultative symbionts from arthropod species able to grow independently from their hosts [63]. These results suggest that the facultative living style may correlate with the phylogeny of the strains. However, further studies are necessary in order to understand the link between phylogeny and lifestyle within this phylogenetic group. Interestingly, strain GP appears to share many conserved genes with *L. chironomi* DSM 19883^T^ [64], a facultative symbiotic bacterium isolated from a member of the *Chironomidae* family (56.49% POCP, Fig. 3c). Bidirectional best-hits (BDBH) analysis with GET_HOMOLOGUES of these two strains showed that they share 1372 orthologs gene clusters (data not shown), amounting to 38.6% of the total CDS of strain GP. Most of these genes are linked to central metabolic pathways. As strain GP, *L. chironomi* also carries iron-heme acquisition operons *hmu*TUV (accessions no. WP_024357741.1, WP_024357742.1 and WP_029747012.1, respectively) and *efe*UOB (accessions no. WP_02436012.1, WP_024356011.1 and WP_024356010.1, respectively), and a homolog of heme oxygenase (*hmu*O, accession no. WP_024356032.1). However, unlike in strain GP, *L. chironomi* does not bear operons *efe*UOB and *hmu*TUV adjacently in its genome. The *efe*UOB operon and *hmu*O are absent from the softcore genome of *Leucobacter* spp., but are shared between several members of this genus (data not shown). Furthermore, strain GP also carries a chromate transport protein A (locus tag D3X82_06990) which was confirmed to be linked to chromate resistance and a common feature shared among several members of the *Leucobacter* genus [65].

### Estimation of gene loss in strain GP

Prior studies suggested that strain GP was obligatorily dependent on *A. denitrificans* PR1 for growth, as no isolated colonies of this organism were recovered after incubation in several conditions [26]. Surprisingly, despite its dependent phenotype, strain GP did not show significant genome reduction as it is commonly reported in symbiotic bacteria [60]. In fact, the number of genes and the genome size of this strain was similar to the ones found in other members of the *Leucobacter* genus (Table 2). These results suggest that, despite the PR1-dependent phenotype, strain GP differs from obligate parasites that, in the process of adapting to their hosts, undergo a process called reductive genome evolution, which results in relatively small genomes (often < 1 Mb) [66, 67]. (Table 3) Comparative genomic analysis of the *Leucobacter* genus revealed that the pangenome consists of 12,998 orthologous gene clusters. The clusters present in at least 90% of the *Leucobacter* spp. (28 of 31 genomes) were used as reference to determine potentially missing genes in the draft genome of strain GP. These results were carefully analyzed and manually curated, due to the high frequency of annotation errors associated with draft assemblies [68, 69]. In this way, of all these clusters, only 141 were present in 90% of the *Leucobacter* spp. and were apparently absent from the draft genome of strain GP. From these 141 clusters, only 9 clusters were non hypothetical genes and no alternative pathways were found in the draft genome of strain GP (Table 3). Among these 9 clusters, only those linked with tetrapyrrole biosynthesis (*hem*ABCE) and thiol transporters (*cyd*DC) may be linked to the incapacity of strain GP to grow independently, as both systems are essential for the synthesis and correct assembly of cytochromes [70–73]. The possible absence of these regions from the genome of strain GP was further investigated by mapping the reads of its MAG against the genome of *L. chironomi* DSM 19883^T^. Visualization of the regions corresponding to these clusters on *L. chironomi* (Table 3) further showed that no reads obtained from strain GP mapped to these regions (see Additional file 1 Figure S5 and S6). The CydDC complex performs the transport of glutathione and L-cysteine and is responsible for maintaining an optimal redox balance in the periplasm [74, 75]. This balance is crucial for the correct assembly of cytochromes in the plasma membrane, and its loss is usually associated with increased sensitivity to high temperature and oxidative stress [71–73]. *hem*ABCE encodes the proteins involved in the synthesis of tetrapyrroles and, subsequently, heme which acts as a prosthetic group in many respiratory and non-respiratory cytochromes [70]. To the best of our knowledge, only a few bacterial strains have been found to be incapable of de novo heme biosynthesis [76]. These strains are mainly pathogenic and affiliated to *Haemophilus influenza*, with the exception of the recently described environmental isolate *Leucobacter* sp. strain ASN212 which requires exogenous heme for growth [76–78]. These organisms rely on complex heme-acquisition systems to thrive in iron-deficient environments and to synthesize essential heme-containing proteins. Functional analysis of the draft genome of strain GP revealed the presence of a heme ABC transport operon (*hmu*TUV) that encodes for a hemin-binding periplasmic protein HmuT (locus tag D3X82_13650), a permease protein HmuU (D3X82_13655) and an ATP-binding protein HmuV (D3X82_13660), respectively. This system has been extensively described and found to be highly conserved in the actinobacterium *Corynebacterium diphtheria* [76]. However, in this organism, additional heme-binding genes (*hta*ABC) and a heme oxygenase *hmu*O were found to be essential for successful heme and iron-heme acquisition [76]. A homolog to *hmu*O was found in the genome of strain GP (locus tag D3X82_07630). However, the conserved *hta*ABC operon, essential for exogenous heme-binding, appeared to be missing. Instead of this operon, strain GP possesses a different adjacent gene cluster encoding for a deferrochelatase/peroxidase EfeB (D3X82_13665), an iron uptake system component EfeO (D3X82_13670) and a ferrous iron permease EfeU (D3X82_13675). These enzymes have been previously linked to ferrous/ferric iron acquisition in *Bacillus subtilis* [79] and intact heme transport in *Escherichia coli* [80]. However, to the best of our knowledge, the EfeUOB system has not been directly linked to intact heme-acquisition in Gram-positive bacteria. In previous studies [26], we have supplied the consortium with exogenous heme and known heme precursors such as coproporphyrin III, coproporphyrin III tetramethylester and coproporphyrin I dihydrochloride, replicating the conditions that allowed the isolation of the heme-dependent *Leucobacter* sp. ASN212 [26, 77]. However, adding these metabolites to agar plates did not abolish the dependent phenotype of strain GP. This result was unexpected as strain GP possesses several downstream genes of the porphyrin pathway; therefore, it should at least be able to use coproporphyrin III as a heme precursor. This finding suggests that either the heme transport system of strain GP is insufficient for intact heme transport across the thick peptidoglycan cell wall or that other essential cofactors or conditions are missing. Unfortunately, the genome of strain ASN212 is not publicly available, which hinders further efforts to characterize the transport of intact heme and heme precursors across the cell wall of this heme-dependent actinobacterium. Considering that strain GP may also lack homologs to thiol ABC transporters (accession numbers WP_024357159.1 and WP_042544611.1), it may be vulnerable to oxidative stress and unable to correctly assemble cytochromes [71–73]. In other sensitive organisms, the absence of this redox regulating system has been compensated by growing deficient strains in catalase-containing media and even in co-culture with catalase-producing bacteria [71, 81]. However, none of these strategies allowed the independent growth of strain GP. Genomic studies are inherently limited because they rely on gene homology for functional annotation and prediction [8]. Furthermore, draft genomes are known to present extensive annotation errors [68], and the presence of a given gene may not even be translated into a particular phenotype. Indeed, genes can be silenced by mutations in the coding region or their promoters, a common phenomenon in the evolution of dependent and pathogenic bacteria which suffer progressive phenotypic and genetic changes due to the interaction with their hosts and the environment [8, 82, 83]. Therefore, these preliminary results require further functional studies in order to understand gene expression and activity in both strains from the microbial consortium.

**Table 3:** Essential genes missing from the draft genome of strain GP identified by core/pangenome analysis with GET_HOMOLOGUES [54]

Representative accession no.				
*L. chironomi* DSM 19883^T^	Strain PR1	KO identifiers	Description	Pathway/System
WP_024356349.1	ASC67664.1	K01476	Arginase RocF	L-arginine biosynthesis; Urea cycle
WP_024355584.1	Absent	K08963, K08964	*S*-methyl-5-thioribose-1-phosphate isomerase MtnA	Methionine salvage pathway
WP_024357159.1	ASC65015.1	K06147, K06148, K16013, K16014	Thiol reductant ABC exporter subunit CydD	Glutathione; L-cysteine ABC transporter
WP_024357158.1	ASC65016.1	K06148, K16012	Thiol reductant ABC exporter subunit CydC	
WP_024356490.1	ASC65168.1	K02492	Glutamyl-tRNA reductase HemA	Porphyrin and chlorophyll metabolism
WP_024356487.1	ASC64797.1	K01698	Porphobilinogen synthase HemB	
WP_084705356.1	ASC63016	K01749	Porphobilinogen deaminase HemC	
WP_024356489.1	ASC64317.1	K01599	Uroporphyrinogen decarboxylase HemE	
WP_024356124.1	ASC67862.1	K02083, K06016	Allantoate deiminase AllC	Purine metabolism

### Unique genes shared between sulfonamide degraders

#### Genetic synteny

**Fig. 5:**
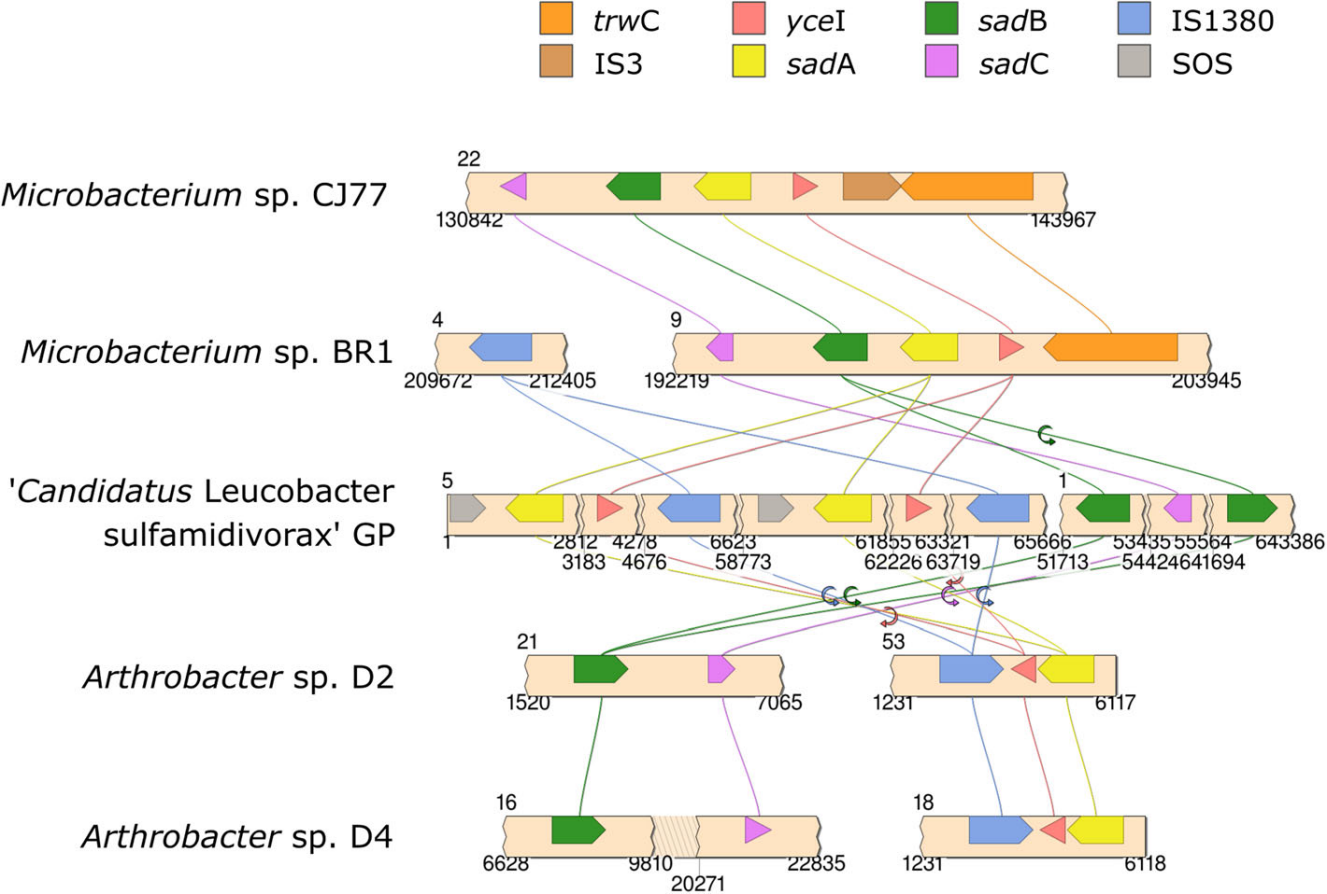
Representation of the genetic organization of the *sad* cluster in sulfonamide degraders: *Microbacterium* sp. strains BR1 and CJ77, *Arthrobacter* sp. strains D2 and D4 and strain GP. Contig numbers and locus for each region are shown next to or on top of the DNA backbone. The diagram was designed with Simple Synteny [85]

As previously discussed, strain GP was shown to be responsible for the breakdown of sulfonamides in the two-member consortium [26]. Some members of the *Microbacterium* genus able to degrade sulfonamides carried a conserved gene cluster encoding for two monooxygenases (SadA and SadB, accession no. WP_100812327.1 and WP_036299419.1, respectively) and one FMN-reductase (SadC, WP_100812326.1). SadA is known to catalyze the *ipso*-hydroxylation of SMX releasing 4-aminophenol, while SadB appears to be responsible for the further oxidation of this unstable and transient metabolite [26, 84]. Although the former enzymes appear to be highly specific for these substrates, the role of SadC can easily be fulfilled by other enzymes with similar activity. This has previously been demonstrated in assays with transformed *E. coli* that contained an incomplete cluster encoding for SadA and SadB alone [84]. The genetic synteny in *Microbacterium* spp. (Fig. 5) appears to be highly conserved [84]. Indeed, all strains of this genus harbor a cluster consisting of a *trw*C relaxase (WP_100812428.1), a polyisoprenoid-binding protein *yce*I (WP_100812427.1), a *sad*A monooxygenase (WP_100812327.1), a *sad*B monooxygenase (WP_036299419.1) and a *sad*C flavin monooxygenase (WP_100812326.1). Except for the putative sulfonamide degrader *Microbacterium* sp. CJ77, that carries an additional gene within the *sad* cluster encoding for an IS3 family transposase (WP_103663393.1), located between *trw*C and *yce*I (Fig. 5). Likewise, strain GP carries homologs for most of these genes, albeit with a different synteny. This strain harbors at least two identical copies of a homolog of *sad*A (locus tag D3X82_17695 and D3X82_17365) in contig 5 (Fig. 5). Both copies of *sad*A are flanked by an SOS response-associated peptidase (D3X82_17690 and D3X82_17360, respectively) and a polyisoprenoid-binding protein *yce*I (D3X82_17700 and D3X82_17370, respectively), which, in turn, is flanked by a single IS1380 family transposase (D3X82_17710 and D3X82_17380, respectively) (Fig. 5).

#### Phylogenetic analysis

**Fig. 6:**
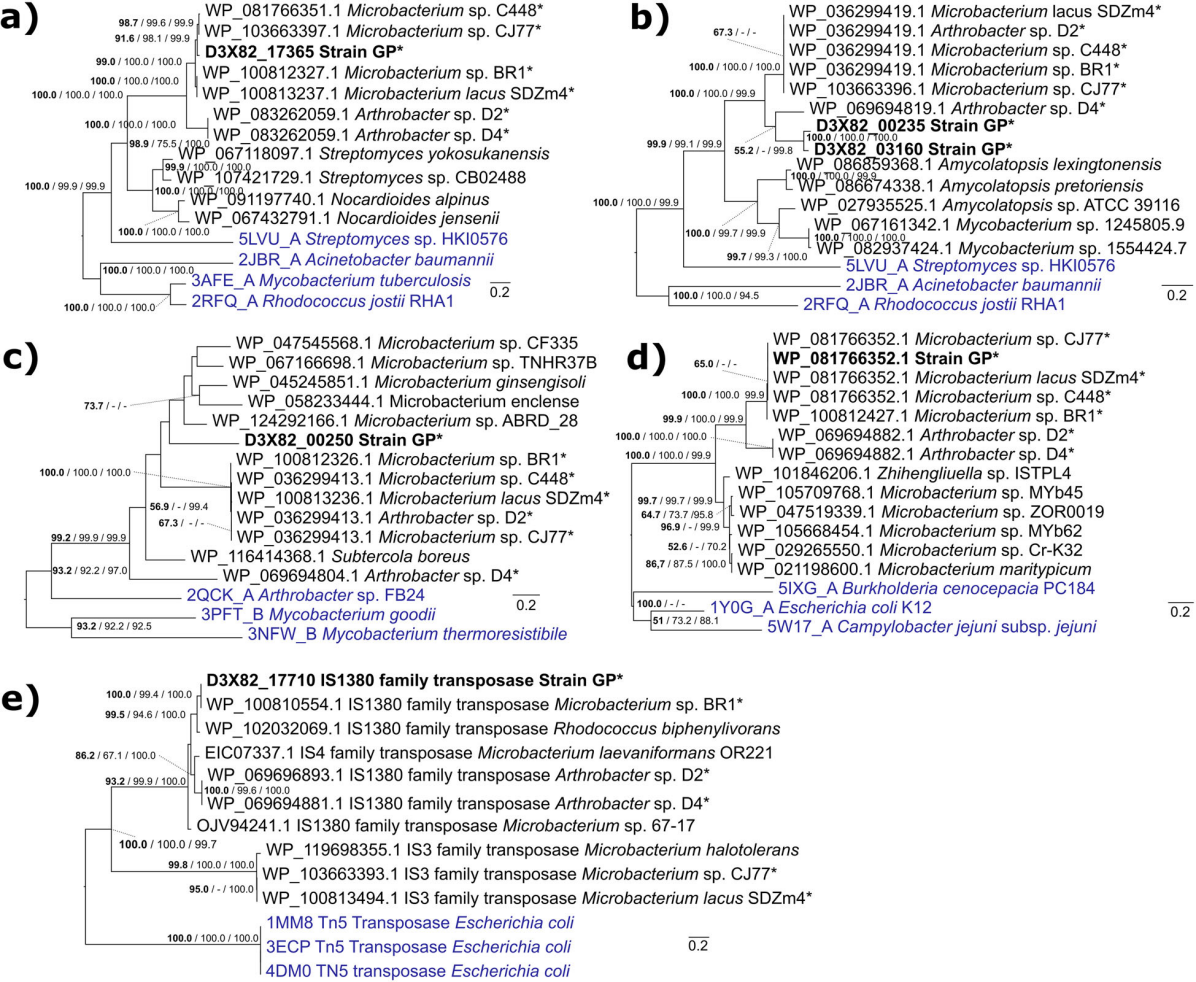
Maximum likelihood phylogenetic trees inferred from amino acid alignments with MEGA6 [87] of (**a**) SadA, (**b**) SadB, (**c**) SadC, (**d**) YceI transporter and (**e**) IS1380/IS3/IS4 transposases shared between sulfonamide degraders. Strain GP is shown in bold; sulfonamide degraders are marked with an asterisk (*); and structural homologs to these enzymes obtained with SWISS-MODEL [88] are shown in bright blue. Node labels indicate: ML bootstrap support above 50% (in bold) / NJ bootstrap support values above 50% / Bayesian posterior probabilities above 70%. The scale bar represents the number of expected substitutions per site. The tree was rooted at the midpoint and visualized with FigTree [125]

The analysis of proteins associated with sulfonamide degradation showed that the SadA and YceI homologs shared a high percentage of amino acid identity between *Microbacterium* sp. and strain GP, while the two *Arthrobacter* sp. isolates were more similar among themselves (see Additional file 1 Figure S7a and d). The high identity between these homologs among strains affiliated to different bacterial species suggests that these genetic determinants may share a common ancestor. This hypothesis was further supported by phylogenetic studies of these proteins. For instance, the Maximum Likelihood (ML) phylogenetic tree shows that all SadA and YceI homologs form a conserved clade with high support values (Fig. 6a and d), also obtained when using both cladistic (Bayesian inference) and distance-based (Neighbor Joining, NJ) methods. Furthermore, the co-existence of both the SadA monooxygenase and the YceI transporter in all the genomes suggests that the YceI binding-protein may play a complementary role in the sulfonamides degradation by enhancing the uptake of these molecules, as previously described for other systems [86]. In contrast, SadB and SadC homologs were highly identical in all *Microbacterium* sp. isolates and *Arthrobacter* sp. D2 (see Additional file 1 Figure S7b and c). And the phylogenetic analysis revealed that these proteins form a highly conserved clade (Fig. 6b and c). Despite the lower identity between the SadB homologs in *Arthrobacter* sp. D4 and strain GP, in comparison to the other sulfonamide-degraders (see Additional file 1 Figure S7b), these proteins also appear to share a common ancestor with their homologs in *Microbacterium* spp. (Fig. 6b). Conversely, the SadC homologs found in these two strains do not appear to share a common ancestor between themselves nor with the other sulfonamide-degrading strains. This result is in agreement with previous studies with the recombinant SadABC complex from *Microbacterium* sp. BR1 [84]. This study showed that SadA and SadB are sufficient to carry out complete SMX degradation in recombinant *E. coli*, suggesting that the role of SadC could be fulfilled by other flavin reductases present in the genome of the host strain [84]. Noticeable, the IS1380 transposase flanking SadA in strain GP is identical to a homolog in *Microbacterium* sp. BR1 (WP_100810554.1, amino acid identity of 100%), and these two proteins form a highly conserved clade in the phylogenetic tree (Fig. 6e). Interestingly, the IS1380 family transposase is located far from the *sad* cluster in *Microbacterium* sp. BR1 (contig 4, instead of contig 9), suggesting that this transposase may be involved in gene mobility in different species and genera of the *Actinobacteria* phylum.

#### Structural analysis of SadA and SadB

**Fig. 7:**
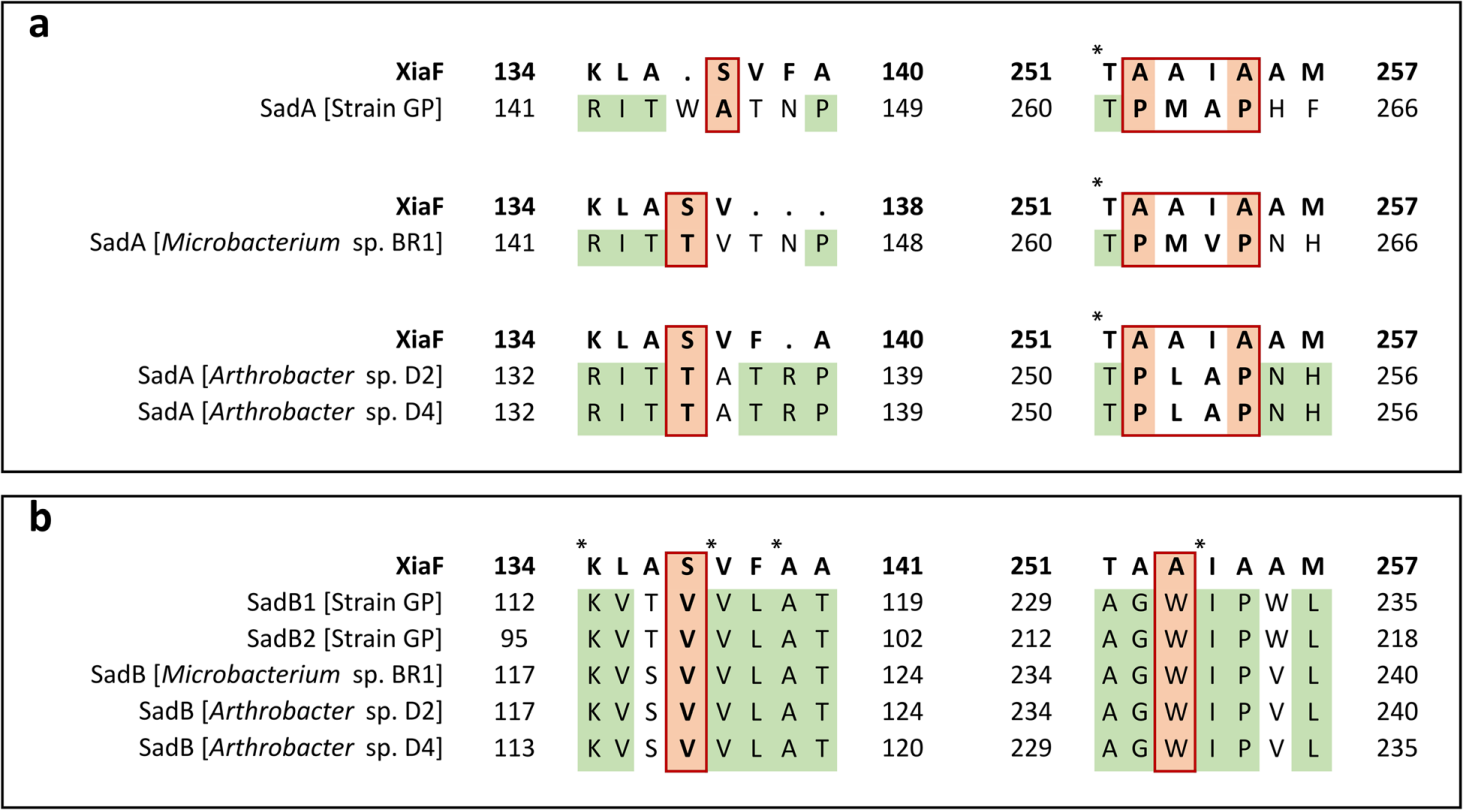
Pairwise alignment with BLASTp of the regions of the substrate binding pocket of XiaF (accession number 5LVW) and each homolog of SadA (**a**) and SadB (**b**) in strains GP, *Microbacterium* sp. BR1 and *Arthrobacter* sp. D2 and D4. Conserved regions between the different SadA and SadB homologs are highlighted in green, nonconserved residues are highlighted in red. Residues shared by all sequences are marked with an asterisk. The diagrams were designed with Excel 2013

Structurally, all SadA and SadB homologs contain an acyl-CoA dehydrogenase domain (see Additional file 1 Figure S8), classifying them as Group D flavoprotein monooxygenases [26, 89]. Furthermore, structural homology search with SWISS-MODEL [88] resulted in highest similarity with XiaF (FADH_2_) from *Streptomyces* sp. HKI0576 (PDB: 5LVW/5LVU), 4-hydroxyphenylacetate hydroxylase (4-HPA) from *Acinetobacter baumannii* (PDB: 2JBR), HsaA monooxygenase from *Mycobacterium tuberculosis* and *Rhodococcus jostii* RHA1 (PDB: 3AFE and 2RFQ, respectively). All of these monooxygenases are known to hydroxylate aromatic compounds. For instance, XiaF is likely involved in terpenoid biosynthesis in *Streptomyces* sp.; it is tetrameric and acts in a two-component system together with a flavin reductase [90]. Furthermore, this monooxygenase can use indole as a surrogate substrate and form indigo and indirubin, as previously described [90], while 4-HPA (EC 1.14.14.9) and HsaA monooxygenases (EC 1.14.14.12) catalyze the insertion of oxygen in the benzene ring of 4-hydroxyphenylacetate or 3-hydroxy-9,10-seconandrost-1,3,5(10)-triene-9,17-dione, respectively. The similarities between XiaF and SadA (32% amino acid identity, 51% similarity, 4% gaps and E-value of 8e-66), SadB1 (locus tag: D3X82_00235, 33% identity, 51% similarity, 2% gaps and E-value of 8e-68), and SadB2 (locus tag: D3X82_03160, 35% identity, 52% similarity, 1% gaps and E-value of 9e-69) are sufficient to suggest a high degree of confidence on the homologous relationship between these proteins [91, 92]. The use of XiaF as a template for modelling resulted in a robust structural prediction of these proteins with quality scores above − 4 (QMEAN, Qualitative Model Energy ANalysis) [93]: − 2.45, − 1.48 and − 1.52, for SadA, SadB1 and SadB2, respectively. These results suggest that the comparison with XiaF is suitable to perform preliminary structural analysis of these monooxygenases. Phylogenetic analysis revealed that XiaF shares a common ancestor with other xenobiotic-degrading enzymes [90], suggesting that both monooxygenases of the SadABC complex likely share ancestry with similar enzymes. However, pairwise sequence alignment revealed a low degree of similarity within the substrate binding pocket of XiaF and homologs regions of SadA and SadB. For instance, the presence of isoleucine I237 in an alpha-helix of XiaF (Fig. 7a) constricts the size of the substrate binding pocket of this monooxygenase. However, alanine residues neighboring I237 are substituted in strains GP, *Microbacterium* sp. BR1 and *Arthrobacter* sp. D2 and D4 by proline residues (P261 and P264 in strain GP) that may induce its structural change to a loop. This conformation probably creates a wider pocket in SadA and may allow easier access to the active site of this monooxygenase. Additional substitutions in the active site of all SadA homologs further support this hypothesis. Specifically, the serine residue S121 (Additional file 1 Figure S7a and Figure S9) of XiaF is substituted by an alanine in strain GP (A145) and threonine in *Microbacterium* sp. BR1 (T144) and both *Arthrobacter* sp. (T135). The serine residue has a hydroxymethyl side chain, while alanine and threonine have a methyl and 1-hydroxyethyl groups, respectively. Serine and threonine are both polar amino acids and likely make the active site of these enzymes amenable for polar cyclic substrates while SadA of strain GP may prefer aromatic substituted substrates. Furthermore, the alanine residue (A145) in the SadA of strain GP would make the active site of this enzyme slightly larger than the active sites of SadA of *Microbacterium* sp. BR1 and XiaF. These findings may explain the differences in SMX degradation rate found between the consortium of strain GP and *A. denitrificans* PR1 and axenic cultures of *Microbacterium* sp. BR1 [94, 95]. For instance, in resting cells conditions the specific degradation rates of the axenic cultures and the consortium were similar (approximately 2 μmol/g_cell dry weight_ x min) [94, 95], however, considering that the abundance of strain GP is significantly low (1–4% relative abundance) [26], this strain appears to be more efficient than *Microbacterium* sp. BR1. Furthermore, in the consortium, 4-aminophenol never accumulated in sufficient amounts to be detected and was only observed in incubations with ^14^C-SMX saturated with an excess of the unlabeled 4-aminophenol [26]. Conversely, the substitutions and subsequent changes in the structure of SadB are harder to predict. For instance, in all SadB homologs, the XiaF S121 is substituted by a valine (isopropyl side chain, Fig. 7b). Furthermore, the A236 of XiaF is substituted by tryptophan in all SadB homologs (W231 in SadB1 of strain GP) suggesting that SadB’s binding pocket could be significantly smaller than XiaF’s and thus accommodate smaller substrates. Despite the low amino acid identity between some of the SadB homologs (see Additional file 1 Figure S7b), the analysis of the conserved domains indicates that the active site could be highly conserved among these enzymes. In this way, although none of the expected metabolites were detected during 4AP degradation in strain GP [26], the presence of homologs of SadB in the genome of this strain suggests that it might catalyze 4AP hydroxylation as previously described for *Microbacterium* sp. BR1 [96].

### Taxonomic classification of strain GP

The total dependency of strain GP on strain PR1 and the lack of similar organisms hinder further efforts for accurate taxonomic classification. Nevertheless, according to the recommendations of the International Committee on Systematic Bacteriology, organisms unable to grow in pure culture can have a provisional taxonomic status (*Candidatus*) [97]. When comparing our data with the standards proposed by Konstantinidis et al. [98] to describe uncultivated prokaryotes and/or those forming microbial consortia, we propose to classify strain GP in a provisional new species within the genus *Leucobacter*, ‘*Candidatus* Leucobacter sulfamidivorax’.

### Description of ‘*Candidatus* Leucobacter sulfamidivorax’

‘*Candidatus* Leucobacter sulfamidivorax’ [sul.fa.mi.di.vo’rax N.L. n. *sulfamidum*, sulfonamide; L. adj. *Vorax* devouring, ravenous, voracious; N.L. masc. Adj. *sulfamidivorax*, sulfonamide-degrading]. Forms a bacterial consortium with *A. denitrificans* strain PR1 and can only be cultured in association with this *Proteobacteria*. Cells stain Gram-positive, present a rod-shaped morphology (1.3 ± 0.03 μm long and 0.5 ± 0.03 μm wide), and are probably non-motile. It produces light yellow-colored colonies with less than 1 mm in diameter on top of the colonies of *A. denitrificans* strain PR1 after 10 d of incubation on 25% (w/v) BHI plates at 30 °C. In liquid medium, it constitutes between 1 and 4% of the total cells. In medium MMSY, the aerobic growth is significantly impaired at 37 °C when compared to that at 22 and 30 °C. Growths well at neutral (pH 7.2) and basic (pH 9.5) pH values when compared to that at pH 5.5. Grows better in oligotrophic media (e.g., MMSY), in comparison to complex and rich medium (e.g., BHI and TSA). Tolerates up to 8% (w/v) NaCl. The DNA G + C content is 69.68 mol%. The representative strain, GP, which degrades sulfonamides, was obtained from a sulfamethoxazole enrichment culture produced from activated sludge from an urban WWTP, in North Portugal, in 2011.

## Conclusions

The genomic analysis showed that strain GP carries at least two copies of *sad*A encoding for the previously described sulfonamide monooxygenase. Both copies are flanked by a single IS1380 family transposase and were found in contig 5 that represents a potential plasmid carried by strain GP. Noticeably, a highly similar *sad*A-containing cluster was also found in the genomes of *Arthrobacter* sp. D2 and D4. All sulfonamide-degrading *Actinobacteria* harbored homologs to *sad*B and *sad*C, nevertheless, in strains GP and *Arthrobacter* sp. D2 and D4 these genes were not in the vicinity of *sad*A and were not associated neither with mobile nor integrative elements. Functional analysis of strain GP genome revealed that this strain may have lost some essential genes, mainly of genes linked to tetrapyrrole biosynthesis and thiol transporters. These results strongly suggest that strain GP may be unable of synthesizing respiratory and non-respiratory cytochromes, essential for aerobic growth, and may need a helper strain to provide exogenous heme and help maintain an optimal redox balance. However, supplying strain GP with exogenous heme and catalase did not abolish this strain’s dependent phenotype. Additional studies are necessary to evaluate the gene expression in strain GP and the mechanisms of intact heme acquisition in this Gram-positive bacterium. Our data suggests that strain GP should be considered as the representative strain of a putatively new species within the *Leucobacter* genus, ‘*Candidatus* Leucobacter sulfamidivorax’.

## Methods

### Culture conditions and DNA extraction

Five type strains of the genus *Leucobacter* were selected for comparative studies based on 16S rRNA gene pairwise similarity to strain GP (Table 1) [26] and purchased from DMSZ (Germany). These strains were grown in Brain-Heart Infusion (BHI, Sigma) for 15 h. All incubations were carried out in the dark at 30 °C under continuous shaking at 120 rpm. The two-member consortium [26] consisting of *Achromobacter denitrificans* PR1 (LMG 30905) and strain GP was incubated for 7 d in mineral medium with 0.2 g/l yeast extract, 4 mM ammonium sulfate, 700 mM succinate, 0.6 mM SMX, and 2.5 g/L 2-phenylethanol (Sigma) as an inhibitor of Gram-negative cells (MMSY-SMX-PE). Further attempts to isolate strain GP were performed by incubating the consortium in 25% BHI agar plates (v/v) with 0.6 mM SMX, heme or heme precursors (10 μg/l, coproporphyrin III, coproporphyrin III tetramethylester, coproporphyrin I dihydrochloride) [26], putrescine (9 μg/l) and catalase (500 U) from Sigma, respectively. Genomic DNA extraction of the *Leucobacter* spp. type strains and the two-member consortium was performed from 2 × 10^10^ cells with GenElute Bacterial Genomic DNA Kit (Sigma) as previously described [26].

### Physiological characterization of the consortium

The effect of environmental parameters on the abundance of each strain of the consortium was investigated. The effect of temperature was examined by incubating the culture in MMSY at 22 °C, 30 °C and 37 °C. The influence of pH was tested at 30 °C in diluted Lysogeny broth medium (DLB, 25% w/v) with 12 mM of MES (pH 5.5), 12 mM phosphate buffer (pH 7.2) or 12 mM of CAPS (pH 9.5) at 30 °C. The tolerance to NaCl was examined in DLB supplemented with NaCl at final concentrations of 2, 4, 6, 8 or 10% (w/v) at 30 °C. To determine the influence of different standard media in the growth of strain GP, the consortium was incubated in unbuffered R2A, TSA and different dilutions of BHI (5, 25, 50, 75 and 100%). Cultures under all these conditions were incubated at 30 °C for 15 h and carried out in triplicate and in parallel to an abiotic control. The abundance of each strain in the consortium was assessed by quantitative PCR with primers targeting the 16S rRNA gene as previously described [26]. Significant differences (*p* < 0.05) between overall abundance of strain GP were determined either by two-way ANOVA (to compare 16S rRNA copies/ml of GP and PR1 at different pH, temperature and salinity) or one-way ANOVA (to compare the ratio of the 16S rRNA copies/ml of strains GP and PR1 across different media) and Tukey’s tests using RStudio v 1.1.463 running with R v3.5.2 [33, 99, 100].

### Electron microscopy

The consortium was visualized in mid-stationary phase (12 h incubation, MMSY, 0.6 mM SMX) by Cryo-Transmission Electron Microscopy (Cryo-TEM) for morphological characterization. Briefly, a 4 μl aliquot of the overnight grown liquid culture was adsorbed onto a holey carbon-coated grid (Lacey, Tedpella, USA), blotted with Whatman 1 filter paper and vitrified into liquid ethane at − 180 °C using a vitrobot (FEI, USA). Frozen grids were transferred onto a Talos Electron microscope (FEI, USA) using a Gatan 626 cryo-holder (GATAN, USA). Electron micrographs were recorded at an accelerating voltage of 200 kV using a low-dose system (20 to 40 e−/Å2) and keeping the sample at − 175 °C. Defocus values were − 3 to 6 μm. Micrographs were recorded on 4 K × 4 K Ceta CMOS camera. The cell size, and periplasmic and cell wall thickness were measured with Fiji from the ImageJ platform [101]. For Transmission Electron Microscopy (TEM) analyses, 4 μl aliquot of the sample was adsorbed onto a glow-discharged carbon film-coated copper grid, and subsequently negatively stained with 2% uranyl acetate. Images were recorded using Philips CM200FEG electron microscope operating at 200 kV on TemCam-F416 CMOS camera (TVIPS, Germany).

### *Leucobacter* spp. type strains whole-genome sequencing and assembly

High-quality DNA of the selected *Leucobacter* spp. type strains (Table 1) was used for paired-end sequencing (2 × 150 bp) with the Hiseq 2500 platform (Illumina) by GATC Biotech (Germany). Paired-end reads were adapter and quality trimmed (≥ Q20) with the BBDuk tool from the BBMap package v35.74 [102]. High-quality reads were used for de novo assembly with SPAdes v3.11.1 [103] with the option –careful. Contigs longer than 500 bp were used further extension with SSPACE v3.0 [104] with recommended settings. All data has been deposited in NCBI under the BioProject accession number PRJNA489769.

### Whole consortium sequencing

The metagenome of the consortium was sequenced in-house in the Miseq (Illumina) and MinION (Oxford Nanopore Technologies, ONT) platforms. The paired-end Miseq library was prepared from 1 μg of high-quality DNA with KAPA HyperPrep Kit (Kapa Biosystems) and TruSeq DNA PCR-free LT Kit library adaptors (Illumina) with a few modifications. Briefly, enzymatic fragmentation of the genomic DNA was increased to 25 min, and ligation was performed for 2 h at 20 °C. Eight cycles of enrichment PCR and size selection for fragments with approximately 500–700 bp was carried out with NucleoMag magnetic beads (Macherey Nagel). Paired-end sequencing (2 × 250 bp) was performed in an Illumina Miseq system (Illumina) with a V2 MiSeq Reagent Kit (500 cycles). Two independent libraries were prepared for MinION long-read sequencing. Both libraries were prepared from 1.5 μg high-quality DNA sheared with a g-TUBE (Covaris) to approximately 8 kb fragments. The libraries were then prepared with the 1D genomic DNA sequencing kit (SQK-LSK108), pooled, loaded with the Library Loading Bead Kit (EXP-LLB001) and sequenced using a flow cell with R9.4 chemistry (FLO-MIN 106, Oxford Nanopore).

### Metagenome-assembled genome (MAG) of strain GP

ONT long reads were adapter trimmed with Porechop v0.2.3 [105]. Illumina paired-end reads were adapter and quality trimmed (≥Q20) with BBDuk from the BBMap package v35.74 [102]. The high-quality paired-end reads were used for hybrid error correction of ONT reads with LoRDEC v0.9 [106]. Resulting long reads were subsequently used for whole-metagenome assembly with Canu v1.7 [107]. Metagenomics contigs were analyzed with SSU finder from CheckM v1.0.11, to determine the amount and affiliation of taxonomic bins present in the metagenome [34]. The metagenome was aligned to the complete genome of strain PR1 (Genbank accession no. CP020917) [35] with BLASTn v2.7.1+ to remove contigs affiliated to the proteobacterium [108] (e-value, identity and hit length threshold cutoffs set to 1e-10, 80 and 30%, respectively). Contigs without significant hits were retrieved from the metagenome and used to construct the new taxonomic bin corresponding to strain GP. Both ONT Illumina-corrected and Illumina reads were used for read binning between strain PR1 and GP with GraphMap v0.5.2 [109] and BWA v0.7.12 [110], respectively. Reads mapping uniquely to strain GP bin were used for hybrid re-assembly with SPAdes v3.7.1 [103]. High-coverage contigs (≥ 1x *k-mer* coverage) obtained with hybrid assembly were used for further scaffolding and polishing with Circlator v1.5.5 [38] and four iterations with Pilon v1.22 [111]. All data has been deposited in NCBI under the Bioproject accession number PRJNA490017.

### Genome annotation, completeness and mobile genetic elements

Quality scores of draft assemblies were assessed with QUAST v4.6.3 [49]. Genome contamination and completeness were determined with CheckM v1.0.11 [34], and tRNA were identified with tRNAscan-SE v2.0 [36]. Open-reading frames (ORFs) were predicted and annotated with NCBI Prokaryotic Genome Annotation Pipeline (PGAP) v4.7 [112] and with RASTtk on the RAST webserver v2.0 [113]. Antibiotic resistance genes were confirmed by aligning amino-acid sequences with BLASTp v2.7.1+ against the Antibiotic Resistance Database (ARDB) v1.1 from July, 2009 [114] and by analyzing the draft genome with the Resistance Gene Identifier (RGI) against the CARD database v3.0.1 [115]. Functional annotation and KEGG Orthology (KO) assignment was further performed with eggNOG-Mapper v4.5.1 [55] and BlastKOALA v2.1 [55]. The presence of plasmids in the genome of strain GP was investigated by assessing differences in coverage and G + C content between contigs, and by further searching for similarities with other plasmids using NCBI BLAST against the non-redundant (*nr*) database on November, 2018 [116]. The differences in coverage were identified by mapping both Illumina and ONT reads against the metagenome of the consortium (concatenated draft assembly of strain GP and complete genome of strain PR1) with BWA v0.7.12 [110] or Graphmap v0.5.2 [109], respectively. The coverage of sorted BAM files was evaluated with Qualimap v2.2.1 [117]. Genes typically associated with plasmids [44] were identified by aligning amino-acid sequences against the CDD database (v3.17) using the NCBI conserved domain search on with default settings on November, 2018 [118–120]. Conjugative elements associated with the type VI secretion systems and possible origins of replication were analyzed with CONJscan v1.0.2 using the Galaxy platform at Pasteur [40–42].

### Phylogenetic analysis of strain GP

The full genome and the 16S rRNA gene of all fully sequenced *Leucobacter* spp. isolates and MAGs were used for phylogenetic analysis (see Additional file 2 Table S3). Sequences were retrieved from the NCBI database (last accessed on November, 2018) [45], except for *Leucobacter* sp. AEAR which was available on GitHub [121]. Moreover, representative genomes of *Microbacterium*, *Leifsonia*, *Agromyces* and *Arthrobacter* genera, were further included to serve as outgroup (see Additional file 2 Table S3). 16S rRNA sequences were used for multiple sequence alignment with MUSCLE in MEGA6 [87, 122]. The phylogenetic tree was inferred from maximum likelihood analyses using MEGA6 [87] with the best-fitting model: Tamura-Nei [123] substitution model with gamma distribution and invariant sites (TN93 + G + I). Bootstrap support values were inferred from 1000 replicates. The PhyloPhlAn pipeline v0.99 [52] was used to infer phylogenomic relationships among fully sequenced members of the *Leucobacter* genus and strain GP (data available on July 2019, see Additional file 2 Table S3). 400 universal proteins were identified and extracted with USEARCH v5.2.32 [53] and used for amino acid alignments with MUSCLE v3.8.31 [122]. The concatenated alignments were used for approximately-maximum-likelihood analysis with FastTree v2.1.8 [124] and the computation of local support values was performed using the Shimodaira-Hasegawa test [53]. Both the 16S rRNA phylogenic tree and the PhyloPhlAn phylogenomic tree were visualized with FigTree v1.4.3 [125] and rooted at the midpoint or the outgroup, respectively. 16S rRNA gene pairwise sequence similarity, Average Nucleotide Identity (ANI), Average Amino-acid Identity (AAI), and Percentage of Conserved Proteins (POCP) [50] between strain GP and the validly named and fully sequenced strains of the *Leucobacter* genus on July 2019 (see Additional file 2 Table S3) were determined using the pairwise similarity tool and 16S-based ID app available on the EzBioCloud platform [126], AAI/ANI-matrix from the enveomics toolbox web server [127] and the POCP.sh script developed by Harris et al. [128] and publicly available on figshare [129]. In addition, the 16S rRNA pairwise sequence similarity, ANI, AAI and POCP values were determined for seven *Leucobacter* strains for which the genome sequences become available during the revision of the present manuscript (see Additional file 2 Table S3).

### *Leucobacter* spp. core and pangenome analysis

Gene search between *Leucobacter* spp. genomes (last accessed on November 2018) and strain GP (see Additional file 2 Table S2) was computed with the GET_HOMOLOGUES package v3.1.4 [54] using all-against-all NCBI BLAST v2.2 with default settings and Pfam-domain scanning. Clustering was performed with COGtriangles v2.1 (−G -t 0 -D), orthoMCL v1.4 (−M -t 0 -D) and BDBH (−D -e) algorithms. For comparison between two genomes only BDBH was used. For the pangenome analysis, the clusters were generated from the intersection of COGtriangles and orthoMCL using the compare_clusters.pl script (−t 0) from the GET_HOMOLOGUES pipeline [54]. To determine core metabolic pathways shared between *Leucobacter* spp. and strain GP, orthologous gene clusters present in the core and softcore genome (100 or 95% of the genomes) [130] were used for functional annotation with eggNOG-Mapper [55] and BlastKOALA [55]. The list of KO identifiers was used to visualize and analyze core metabolic pathways in KEGG [131] and compared to the metabolic reconstruction obtained with Pathway Tools v22.0 [132].Conversely, to evaluate possible gene loss the analysis was carried out in three different stages: (i) detection, (ii) manual curation and (iii) mapping of the metagenomics reads against a closely related *Leucobacter* spp. genome. In the first step, we applied loose criteria and determined which clusters were present in at least 90% (28 of 31 genomes) of *Leucobacter* spp. genes but absent from the genome of strain GP. The clusters were found by analyzing the pangenome with the parse_pangenome_matrix.pl script from the GET_HOMOLOGUES pipeline [54]. Loose criteria instead of tight criteria (i.e. genes from the core genome) was chosen for this first stage because the vast majority of the sequences used in this analysis originated from draft genomes, which may contain significant gaps and annotation errors. In the second stage, clusters marked as missing were manually curated to exclude annotation errors in the genome of strain GP. This was done by aligning representative sequences of each cluster to the draft genome of strain GP with NCBI tBLASTn [116]. Moreover, in the third stage, metagenomics reads obtained from Miseq sequencing were binned between *A. denitrificans* PR1 and a reference genome affiliated to the *Leucobacter* genus – *L. chironomi* DSM 19883^T^ – using BBSplit from the BBMap package v35.74 [102]. Alignments of strain’s GP reads against the reference genome of *L. chironomi* were inspected in the Integrative Genomics Viewer (IGV) v2.6.3 [133] to further determine the presence or absence of high quality reads mapping against essential missing regions.

### Whole genome comparisons and evolution of the SadABC complex

Whole genome comparison between strain GP and other sulfonamide degraders (see Additional file 2 Table S3), was performed with the GET_HOMOLOGUES package as described above [54]. The core and softcore genes shared between these strains were obtained by computing the intersection of clusters generated by COGtriangles and orthoMCL using the compare_clusters.pl script (−t 7; −t 6, respectively). Relevant gene clusters (i.e., *sad*A, *sad*B, *sad*C, *yce*I, and IS1380/IS3/IS4 transposases) were further used for homology searches with BLASTp against the NCBI non-reductant database [116], structural modeling in SWISS-MODEL [88] and conserved domain searches in NCBI database [134]. The phylogeny and evolution of these proteins and their corresponding homologs were inferred from amino acid alignments with MUSCLE in MEGA6 [87, 122]. The phylogeny was estimated from combination of three methods: Maximum Likelihood (ML), Bayesian optimization and Neighbor Joining (NJ). For the ML method, the amino acid alignments were first evaluated with ProtTest v3.4.2 [135] to find the best-fitting model of protein substitution. For SadA and SadB phylogeny the LG substitution model was used [136] with gamma plus invariant sites heterogeneity model (G + I); for SadC and YceI the WAG model [137] was used with G or G + I, respectively; and for the transposase the JTT model [138] was used with the G heterogeneity model. The ML trees with bootstrap support values from 1000 replicates were constructed with MEGA6 [87]. Bayesian optimization was calculated with BEAST v1.10.4 [139]. Markov chain Monte Carlo (MCMC) were run using one million iterations and trees were sampled every 100 generations. The results of triplicate runs were combined with LogCombiner from the BEAST package [139], and the combined output was analyzed with Tracer v1.7.1 to assess the overall quality of the estimation [140]. Posterior probability support values and consensus tree was calculated from 10% of the total number of iterations (300,000). For the NJ method, the phylogenetic trees were constructed in MEGA6 using the JTT model [138] with uniform rates and bootstrap support values were inferred from 1000 replicates. The ML trees were rooted at midpoint and visualized with FigTree v1.4.3 [125], Bayesian posterior probability values, and ML and NJ bootstrap support values were included in the final tree. Functional comparison between strain GP and its helper strain, *A. denitrificans* PR1, was performed by submitting both genomes for annotation with RASTtk [113]. Furthermore, the metabolic reconstruction and comparison between these distantly related strains was achieved with the function based comparison tool in the SEED viewer v2.0 [141].

## Supplementary information

**Additional file 1: Figure S1.** Fluorescence microscopy composite images of DAPI-stained cells of the microbial consortium (blue) and (A) cells hybridized with the modified ActORD1 FISH probe (stains strain GP, 5′ fluorophore: FAM, green, sequence: 5’- CACCAGGAATTCCAATCTCC-3’, original probe accession number: pB-1931, reference: [147]) or with (B) cells hybridized with Alca2 FISH probe (stains strain PR1; 5’ fluorophore: Cy3, orange/red; sequence: 5’- CATCTTTCTTTCCGAACCGC-3’; probe accession number: pB-2127, reference: [148]) **Figure S2:** Electron micrographs of negatively stained *Achromobacter denitrificans* PR1 showing the absence heatmap representation of peritrichous flagella(FG) **Figure S3:** Cladogram of the 16S rRNA gene inferred from maximum likelihood estimation with MEGA6 with the best-fitting model: TN93+G+I [87]. *Leucobacter* spp. strains sequenced in this study are marked with an asterisk, and sulfonamide degraders are shown in bold. The tree was rooted at the outgroup and visualized with FigTree [125]. The scale bar represents the number of expected substitutions per site. Bootstrap values were inferred from 1000 replicates, values above 70% are shown at the corresponding nodes **Figure S4:** Presence/absence heatmap representation and dendrograms of the 12,998 orthologs gene clusters found in the pangenome of Leucobacter spp. and strain GP obtained with the GET_HOMOLOGUES package [54]. Each column represents a different gene cluster which can be absent (white) or present (blue) in each strain. As paralogs were included in the analysis, some clusters have more than one homolog per genome, and these are shown in darker blue **Figure S5:** Visualization of the reads of the strain GP’s MAG on the Interactive Genomics Viewer (IGV) [133] mapping to the reference genome and annotations of *Leucobacter chironomi* strain DSM 19883 ^T^ (assembly accession number GCA_000421845.1). This region from strain DSM 19883 (ATXU0100005.1:1..268438) contains the genes from the purine de novo biosynthetic pathway and the porphyrin and chlorophyll metabolism pathway (left to right): phosphoribosylformylglycinamidine synthase subunit PurQ (accession no. WP_017883592.1, locus tag H629_RS0106495); porphobilinogen synthase HemB (accession no. WP_024356487.1, locus tag H629_RS0106505); porphobilinogen deaminase HemC (accession no. WP_084705356.1, locus tag H629_RS14980); uroporphyrinogen decarboxylase HemE (accession no. WP_024356489.1, locus tag H629_RS0106525); glutamyl-tRNA reductase HemA (accession no WP_024356490.1, locus tag H629_RS0106530) **Figure S6:** Visualization of the reads of the strain GP’s MAG on the IGV [133] mapping to the reference genome and annotations of *Leucobacter chironomi* strain DSM 19883 ^T^ (assembly accession number GCA_000421845.1). This region from strain DSM 19883 (ATXU01000008.1:1..186096) contains the genes related to amino acid metabolism and from the glutathione and L-cysteine ABC transporter pathway (left to right): leucine--tRNA ligase (accession no. WP_017793981.1, locus tag H629_RS0110150); alpha/beta hydrolase (accession no. WP_010837840.1, locus tag H629_RS011055); thiol reductant ABC exporter subunit CydC (accession no. WP_024357158.1, locus tag H629_RS0110165); thiol reductant ABC exporter subunit CydD (accession no. WP_024357159.1, locus tag H629_RS0110170) **Figure S7:** Heatmaps representing amino acid identity (BLASTp) of the SadABC (a, b and c) complex and YceI transporter (d) among isolates from the *Microbacterium* genus (strains BR1, C488, SDZm4 and CJ77), *Arthrobacter* genus (strains D2 and D4) and strain GP **Figure S8:** Amino acid alignment with MUSCLE [122] of Acyl-CoA domains: Nterminal (a), middle (b) and C-terminal (c); between SadA and SadB homologs in *Microbacterium* sp. BR1, *Arthrobacter* sp. D2 and D4 and strain GP (SadB1: D3X82_00235; SadB2: D3X82_03160). Conserved regions within SadA and SadB and highlighted in green and conserved regions shared between all proteins are marked with an asterisk **Figure S9:** Close-up of the substrate-binding pocket of XiaF (PDB: 5LVW) bound to FADH and indole obtained by Kugel et al. [90]. FADH is the co-factor, indole the substrate and S121 and I237 are the residues that are modified in SadA of Microbacterium sp. BR1 and strain GP. The ribbon (a) and electrostatic surface potential (b) diagrams have been prepared with PyMol [149]. In b negative potential is shown in red and positive potential in blue.

**Additional file 2: Table S1.** Mean coverage and GC content per strain and contig in the metagenome assembly of the consortium consisting of *Achromobacter* denitrificans PR1 and *Candidatus* Leubacter sulfamidivorax’ **Table S2:** Results from CheckM evalution of the draft assembly of the ‘*Candidatus* Leucobacter sulfamidivorax’ **Table S3:** List of all bacterial strain used for comparative genomics (^T^) type stain; (*) sulfanomide degraders; N.A not available; (bold) strains sequenced in this study;(^1^) available on Github [1]; * the 16S rRNA gene sequence of this strain has a gap between positions 706 and 761;** no rRNA was annotated in this sequence; cells highlighted in orange indicate strain for which the genome sequence became available after November 2018, and, therefore were not included in the comparative genomics studies to assess gene loss in strain GP **Table S4:** Complete and near complete (1 block missing = 1 ortholog gene missing) modules of the softcore genome of *Leucobacter* spp. and strain GP reconstructed in silico with KEGG Mapper [56]

## Abbreviations

AAI: Average Amino Acid Identity
ANI: Average Nucleotide Identity
BHI: Brain-Heart Infusion
Cryo-TEM: Cryo-Transmission Electron Microscopy
DLB: 25% Diluted Lysogeny Broth
FISH: Fluorescent in situ hybridization
FMN: Flavin Mononucleotide
MAG: Metagenome-Assembled Genome
MCMC: Markov chain Monte Carlo
ML: Maximum Likelihood
MMSY: Mineral medium with ammonium sulfate, succinate and yeast extract
MMSY-SMX-PE: MMSY with sulfamethoxazole and 2-phenylethanol
MOB: Mobility element (relaxase)
MPF: Mating-pair formation
MTA: 5′-methylthioadenosine
NJ: Neighbor Joining
ONT: Oxford Nanopore Technologies
POCP: Percentage of Conserved Genes
R2A: Reasoner’s 2A Medium
RAxML: Randomized Accelerated Maximum Likelihood
T4CP: Type IV coupling protein
T4SS: Type IV secretion system
TEM: Transmission Electron Microscopy
TSA: Tryptic Soy Broth
