# Supplementary material for: Comparative genomics reveals a novel genetic organization of the sad cluster in the sulfonamide-degrader ‘Candidatus Leucobacter sulfamidivorax’ strain GP
Source: BMC Genomics. 2019 Nov 21;20:885. doi: 10.1186/s12864-019-6206-z (PMC6868719; doi:10.1186/s12864-019-6206-z)
Supplement: Supplementary file 1 — Additional file 1: Figure S1. Fluorescence microscopy composite images of DAPI-stained cells of the microbial consortium (blue) and (A) cells hybridized with the modified ActORD1 FISH probe (stains strain GP, 5′ fluorophore: FAM, green, sequence: 5’- CACCAGGAATTCCAATCTCC-3’, original probe accession number: pB-1931, reference: [147]) or with (B) cells hybridized with Alca2 FISH probe (stains strain PR1; 5’ fluorophore: Cy3, orange/red; sequence: 5’- CATCTTTCTTTCCGAACCGC-3’; probe accession number: pB-2127, reference: [148]) Figure S2: Electron micrographs of negatively stained Achromobacter denitrificans PR1 showing the absence heatmap representation of peritrichous flagella(FG) Figure S3: Cladogram of the 16S rRNA gene inferred from maximum likelihood estimation with MEGA6 with the best-fitting model: TN93+G+I [87]. Leucobacter spp. strains sequenced in this study are marked with an asterisk, and sulfonamide degraders are shown in bold. The tree was rooted at the outgroup and visualized with FigTree [125]. The scale bar represents the number of expected substitutions per site. Bootstrap values were inferred from 1000 replicates, values above 70% are shown at the corresponding nodes Figure S4: Presence/absence heatmap representation and dendrograms of the 12,998 orthologs gene clusters found in the pangenome of Leucobacter spp. and strain GP obtained with the GET_HOMOLOGUES package [54]. Each column represents a different gene cluster which can be absent (white) or present (blue) in each strain. As paralogs were included in the analysis, some clusters have more than one homolog per genome, and these are shown in darker blue Figure S5: Visualization of the reads of the strain GP’s MAG on the Interactive Genomics Viewer (IGV) [133] mapping to the reference genome and annotations of Leucobacter chironomi strain DSM 19883 T (assembly accession number GCA_000421845.1). This region from strain DSM 19883 (ATXU0100005.1:1..268438) contains the genes from the purine de novo [file 12864_2019_6206_MOESM1_ESM.pdf]

Supplementary Figures

Comparative genomics reveals a novel genetic organization of the *sad* cluster in the sulfonamide-degrader '*Candidatus* Leucobacter sulfamidivorax' strain GP

Ana C. Reis[a,b], Boris A. Kolvenbach[b], Mohamed Chami[c], Luís Gales[d,e,f], Conceição Egas[g,h],

Philippe F.-X. Corvini[b], Olga C. Nunes[a]*

[a]LEPABE – Laboratory for Process Engineering, Environment, Biotechnology and Energy, Faculty of Engineering, Department of Chemical Engineering, University of Porto, Rua Dr. Roberto Frias, 4200-465, Porto, Portugal

[b]Institute for Ecopreneurship, School of Life Sciences, University of Applied Sciences Northwestern Switzerland, Gruendenstrasse 40, 4132 Muttenz, Switzerland

[c]BioEM lab, Biozentrum, University of Basel, CH-4058 Basel, Switzerland

[d]i3S – Instituto de Investigação e Inovação em Saúde, Rua Alfredo Allen, 208, 4200-135 Porto, Portugal

[e]IBMC – Instituto de Biologia Molecular e Celular, Rua Alfredo Allen, 208, 4200-135 Porto, Portugal

[f]ICBAS – Instituto de Ciências Biomédicas Abel Salazar, Rua de Jorge Viterbo Ferreira 228, 4050-313 Porto, Portugal

[g]Next Generation Sequencing Unit, Biocant, BiocantPark, Núcleo 04, Lote 8, 3060-197 Cantanhede, Portugal

[h]Center for Neuroscience and Cell Biology, University of Coimbra, 3004-504 Coimbra, Portugal

* Corresponding author: Olga C. Nunes: tel.: +351 225081917; e-mail address: opnunes@fe.up.pt

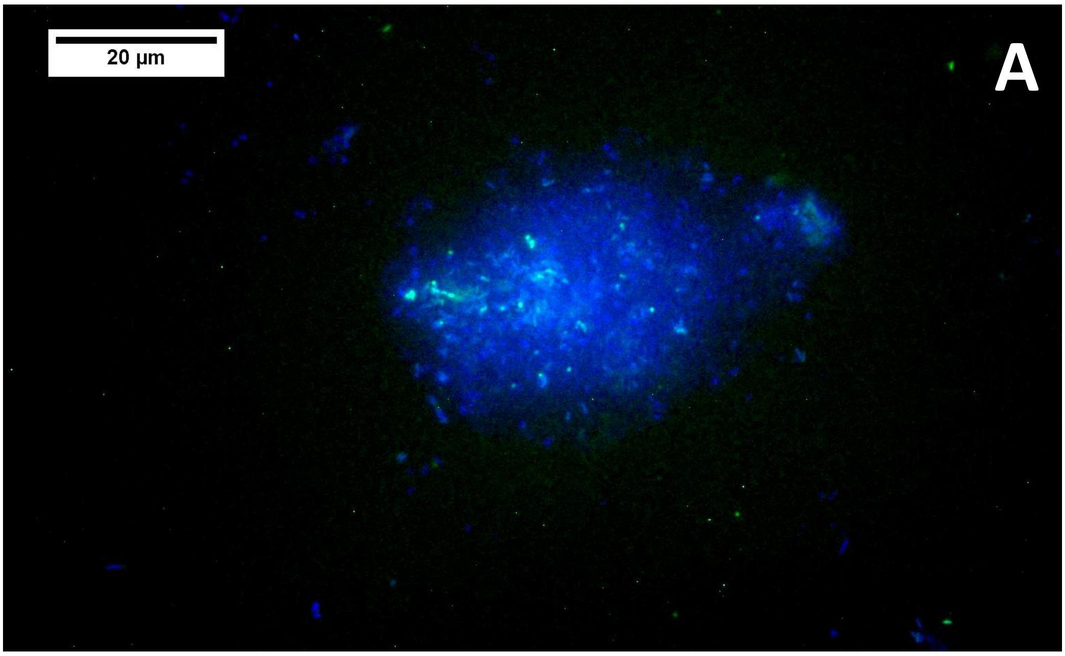

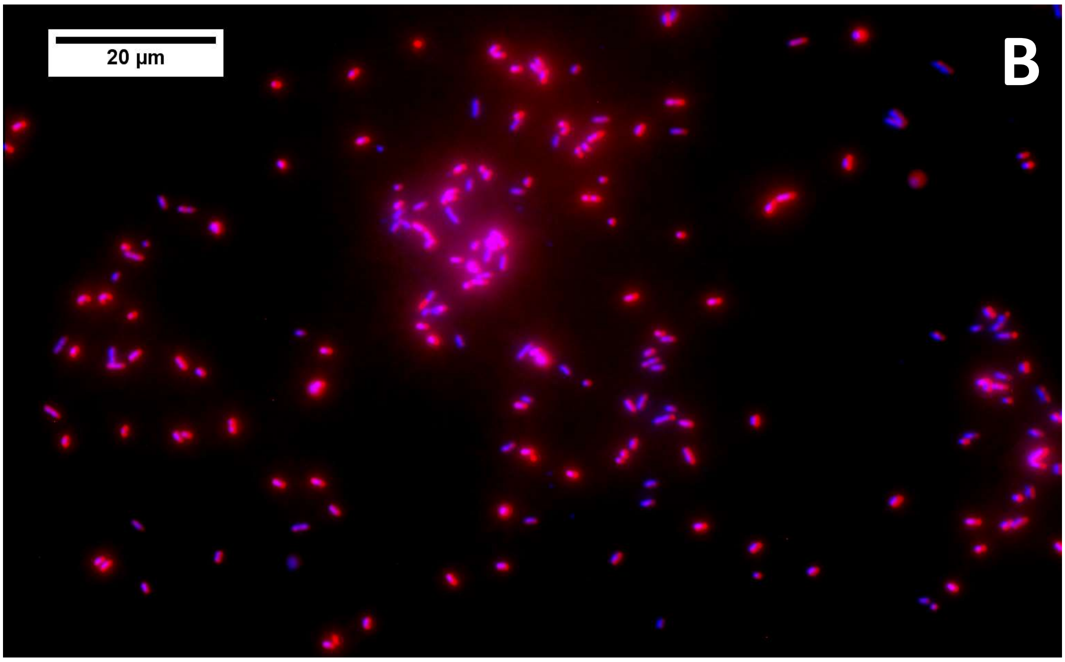

Supplementary Figure 1 – Fluorescence microscopy composite images of DAPI-stained cells of the microbial consortium (blue) and (A) cells hybridized with the modified ActORD1 FISH probe (stains strain GP, 5' fluorophore: FAM, green, sequence: 5'- CACCAGGAATTCCAATCTCC-3', original probe accession number: pB-1931, reference: [1]) or with (B) cells hybridized with Alca2 FISH probe (stains strain PR1; 5' fluorophore: Cy3, orange/red; sequence: 5'- CATCTTTCTTTCCGAACCGC-3'; probe accession number: pB-2127, reference: [2]).

Fluorescence in situ hybridization (FISH) was performed as previously described [3] with a few modifications. To analyze strain GP (A) the cells of the microbial consortium were fixed with ethanol for 16 h at 4 °C. Prior to hybridization the cells were pre-treated with achromopeptidase (Sigma) at 60 U/ml for 60 seconds at 37 °C. While, to analyze *Achromobacter denitrificans* PR1 cells (B) the microbial consortium was fixed with 4 % (v/v) formaldehyde (Sigma) for 3 h at 4 °C and no pre-treatment was performed. Probe concentration was 3 or 10 ng/µl for strain PR1 and GP, respectively. Formamide (Sigma) concentration was 25 or 50% (v/v) for PR1 and GP, respectively.

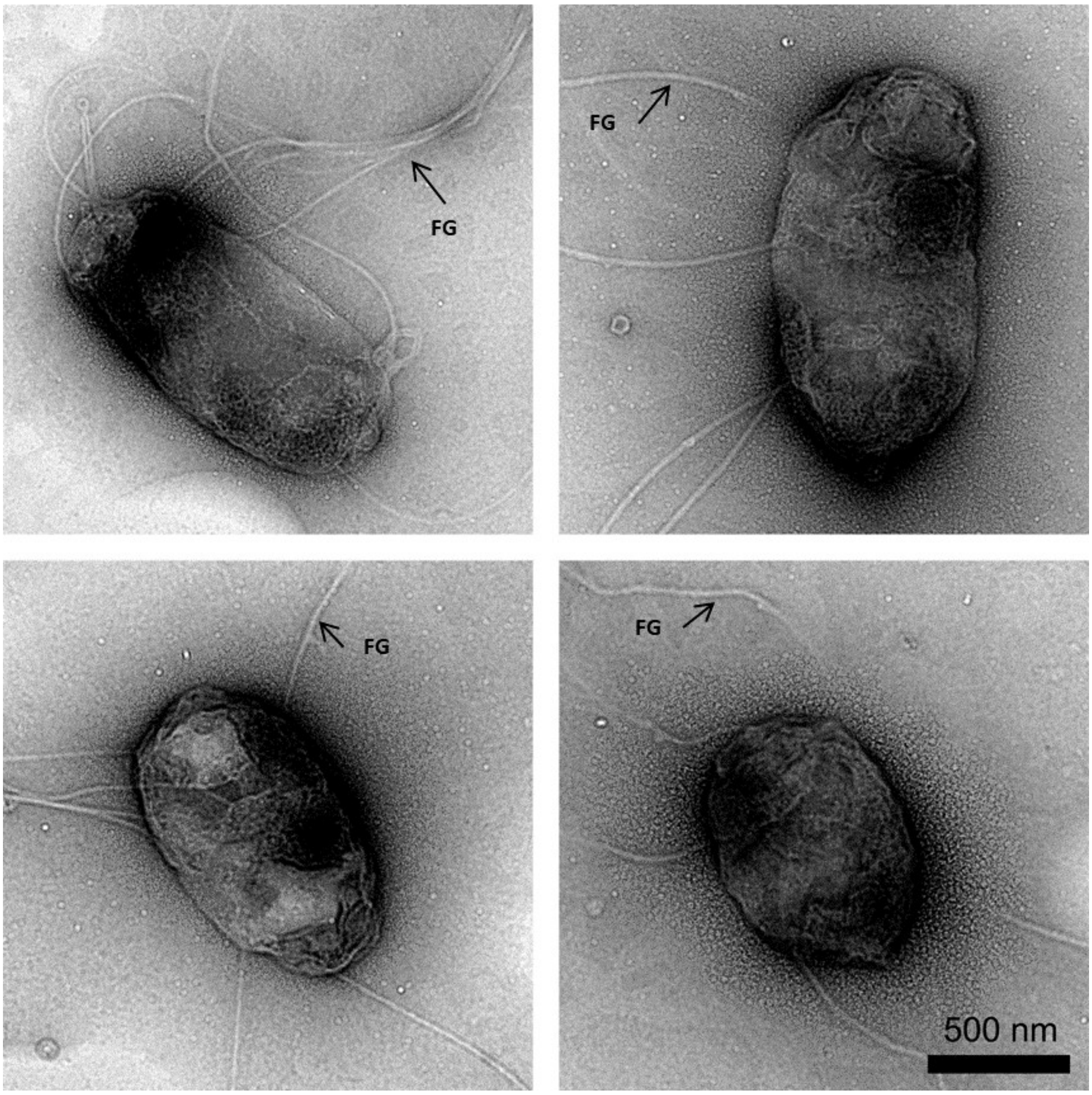

Supplementary Figure 2 –Electron micrographs of negatively stained *Achromobacter denitrificans* PR1 showing the presence of peritrichous flagella (FG).

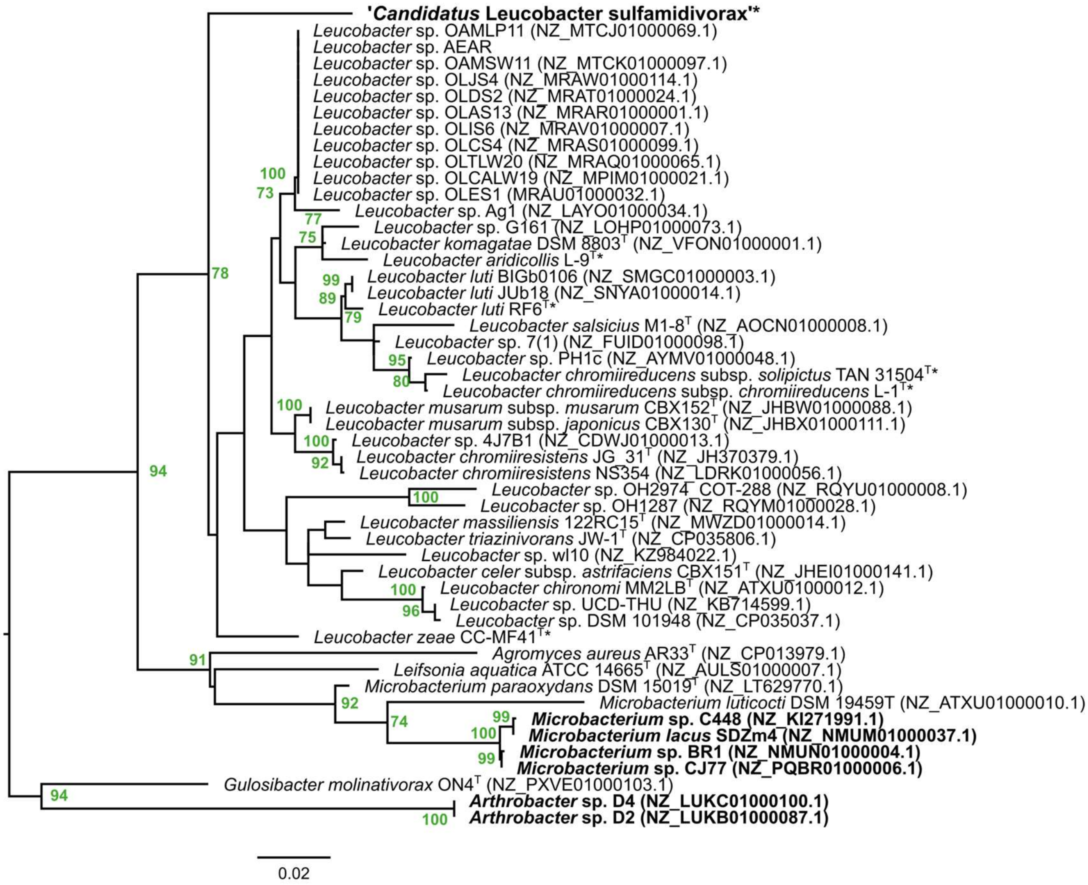

Supplementary Figure 3 – Cladogram of the 16S rRNA gene inferred from maximum likelihood estimation with MEGA6 with the best-fitting model: TN93+G+I [4]. *Leucobacter* spp. strains sequenced in this study are marked with an asterisk, and sulfonamide degraders are shown in bold. The tree was rooted at the outgroup and visualized with FigTree [5]. The scale bar represents the number of expected substitutions per site. Bootstrap values were inferred from 1000 replicates, values above 70% are shown at the corresponding nodes.

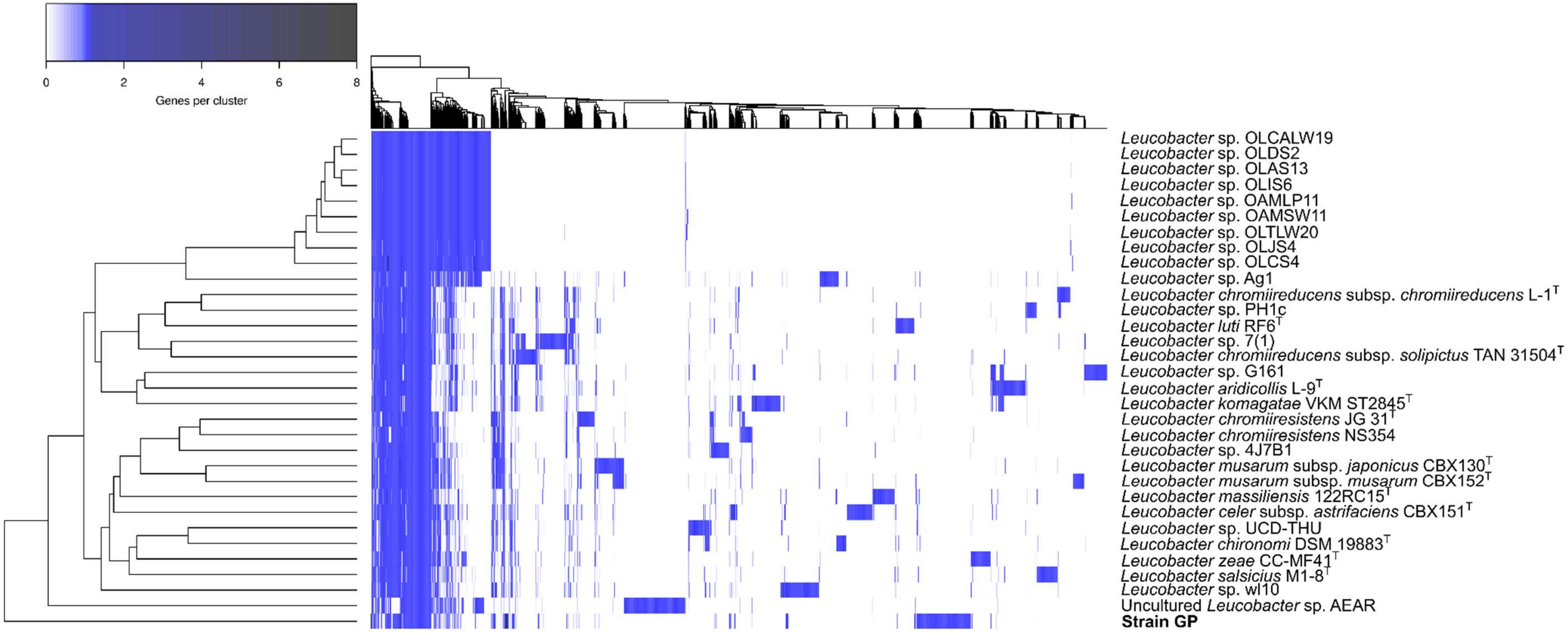

Supplementary Figure 4 – Presence/absence heatmap representation and dendrograms of the 12,998 orthologs gene clusters found in the pangenome of *Leucobacter* spp. and strain GP obtained with the GET_HOMOLOGUES package [6]. Each column represents a different gene cluster which can be absent (white) or present (blue) in each strain. As paralogs were included in the analysis, some clusters have more than one homolog per genome, and these are shown in darker blue.

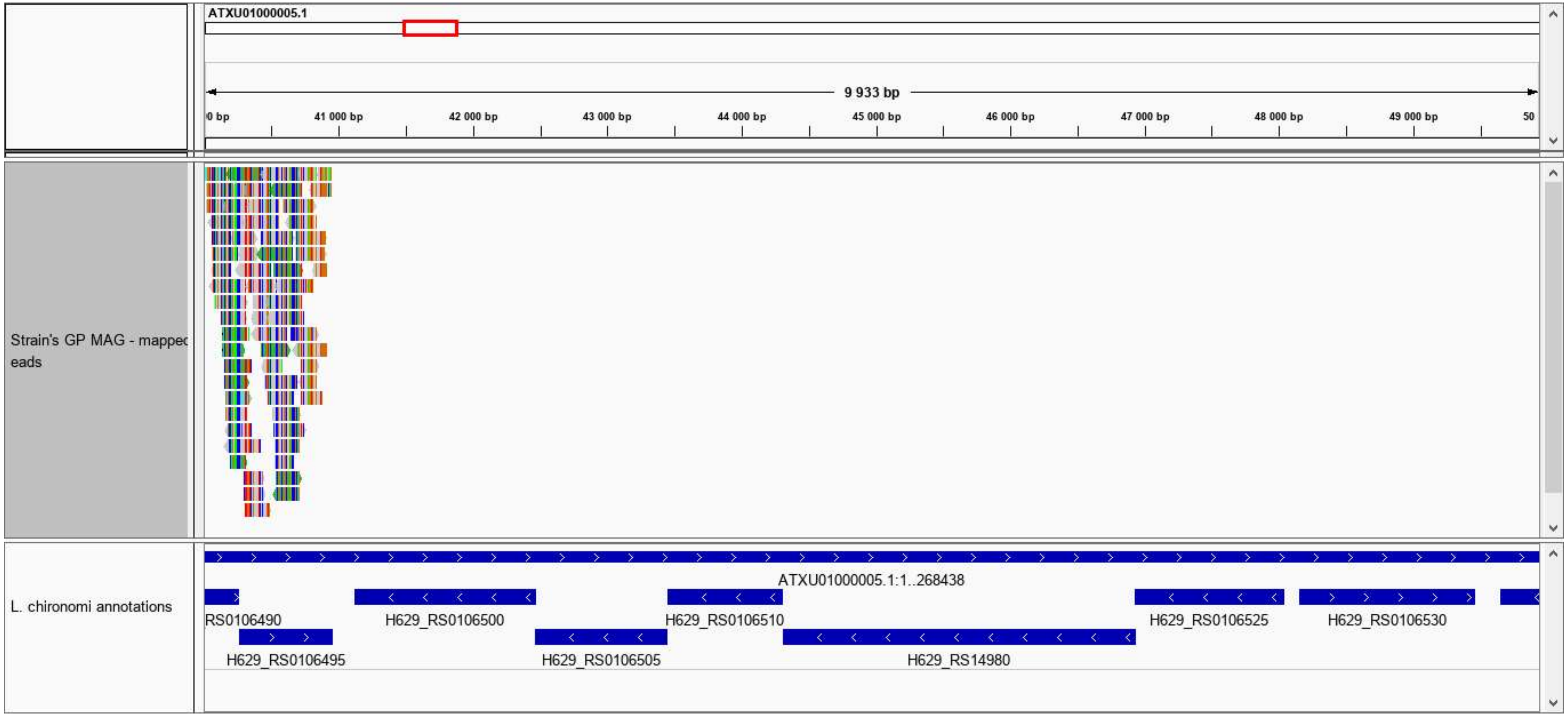

Supplementary Figure 5 – Visualization of the reads of the strain GP's MAG on the Interactive Genomics Viewer (IGV) [7] mapping to the reference genome and annotations of *Leucobacter chironomi* strain DSM 19883^T (assembly accession number GCA_000421845.1). This region from strain DSM19883^T (ATXU0100005.1:1..268438) contains the genes from the purine *de novo* biosynthetic pathway and the porphyrin and chlorophyll metabolism pathway (left to right): phosphoribosylformylglycinamidine synthase subunit PurQ (accession no. WP_017883592.1, locus tag H629_RS0106495); porphobilinogen synthase HemB (accession no. WP_024356487.1, locus tag H629_RS0106505); porphobilinogen deaminase HemC (accession no. WP_084705356.1, locus tag H629_RS14980); uroporphyrinogen decarboxylase HemE (accession no. WP_024356489.1, locus tag H629_RS0106525); glutamyl-tRNA reductase HemA (accession no. WP_024356490.1, locus tag H629_RS0106530).

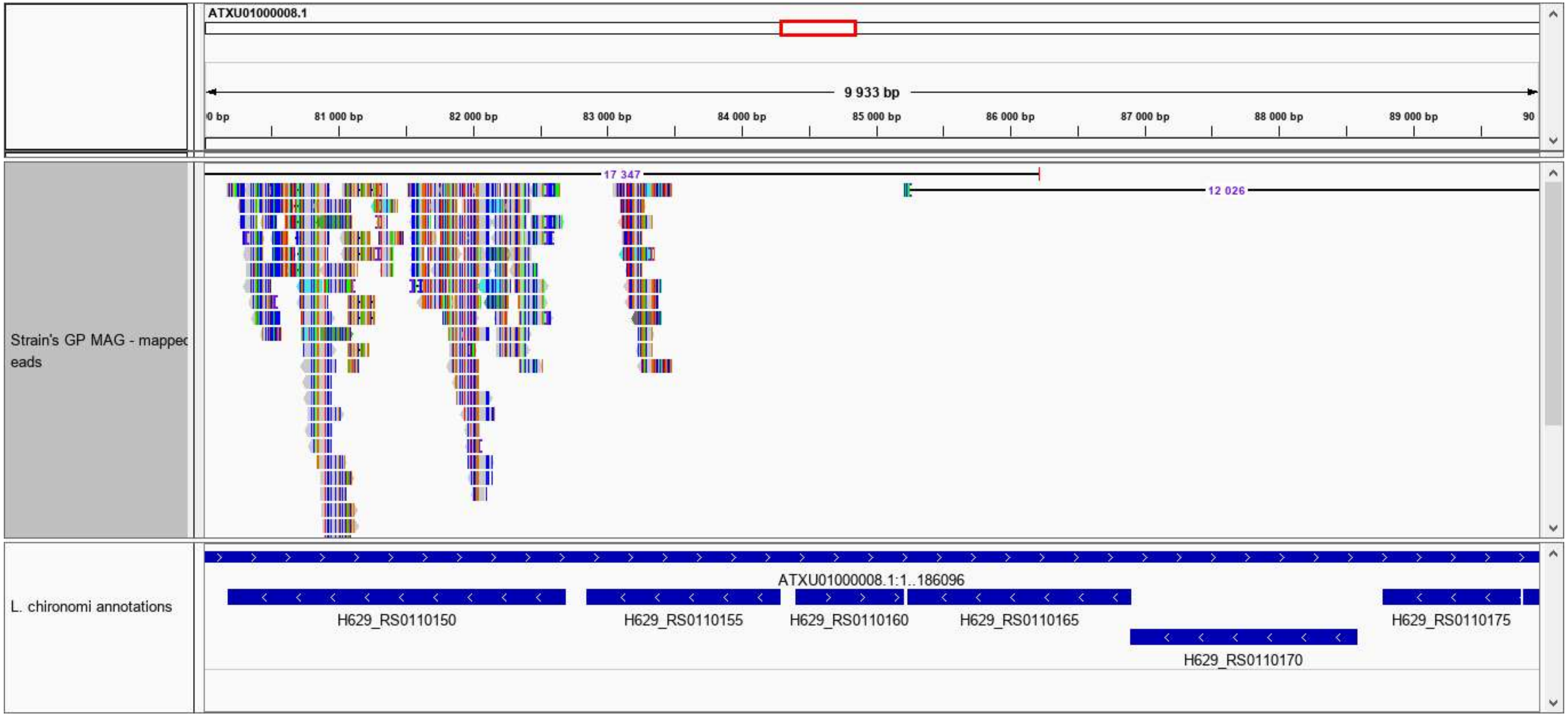

Supplementary Figure 6 – Visualization of the reads of the strain GP's MAG on the IGV [7] mapping to the reference genome and annotations of *Leucobacter chironomi* strain DSM 19883^{T} (assembly accession number GCA_000421845.1). This region from strain DSM19883^{T} (ATXU01000008.1:1..186096) contains the genes related to amino acid metabolism and from the glutathione and *L*-cysteine ABC transporter pathway (left to right): leucine--tRNA ligase (accession no. WP_017793981.1, locus tag H629_RS0110150); alpha/beta hydrolase (accession no. WP_010837840.1, locus tag H629_RS011055); thiol reductant ABC exporter subunit CydC (accession no. WP_024357158.1, locus tag H629_RS0110165); thiol reductant ABC exporter subunit CydD (accession no. WP_024357159.1, locus tag H629_RS0110170).

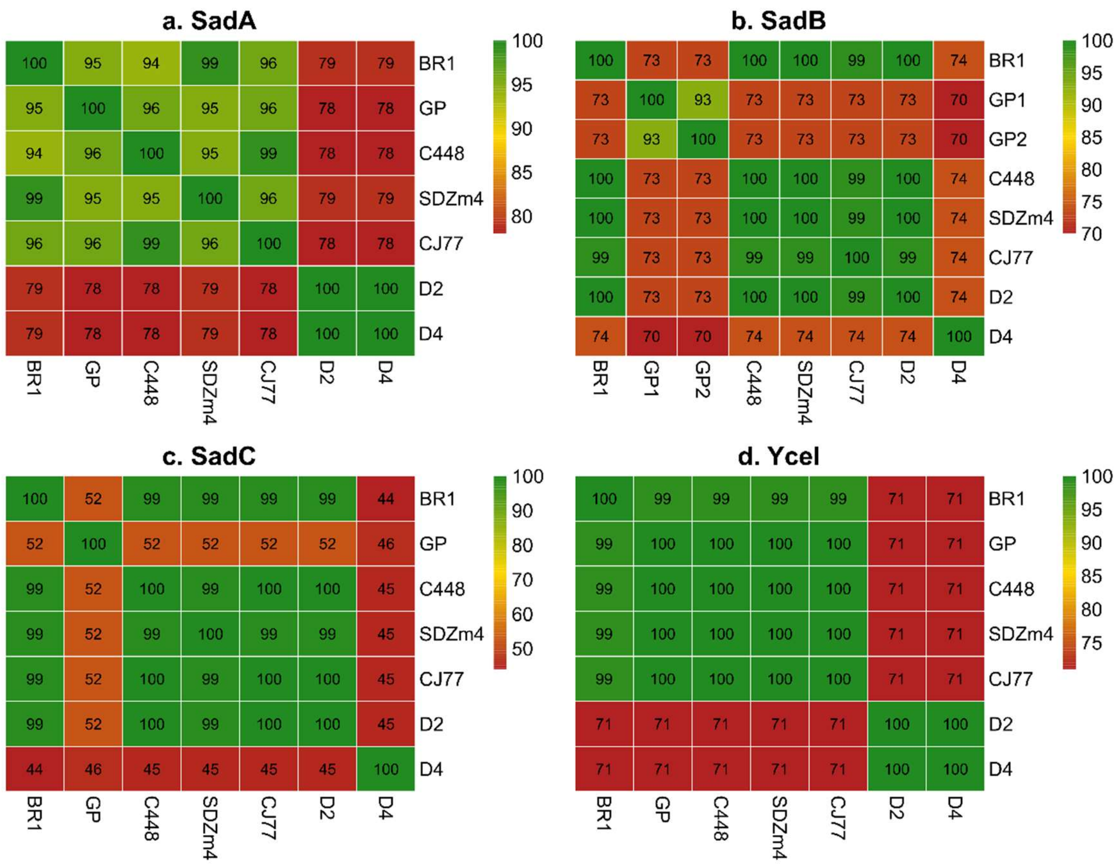

Supplementary Figure 7 – Heatmaps representing amino acid identity (BLASTp) of the SadABC (a, b and c) complex and YceI transporter (d) among isolates from the *Microbacterium* genus (strains BR1, C488, SDZm4 and CJ77), *Arthrobacter* genus (strains D2 and D4) and strain GP.

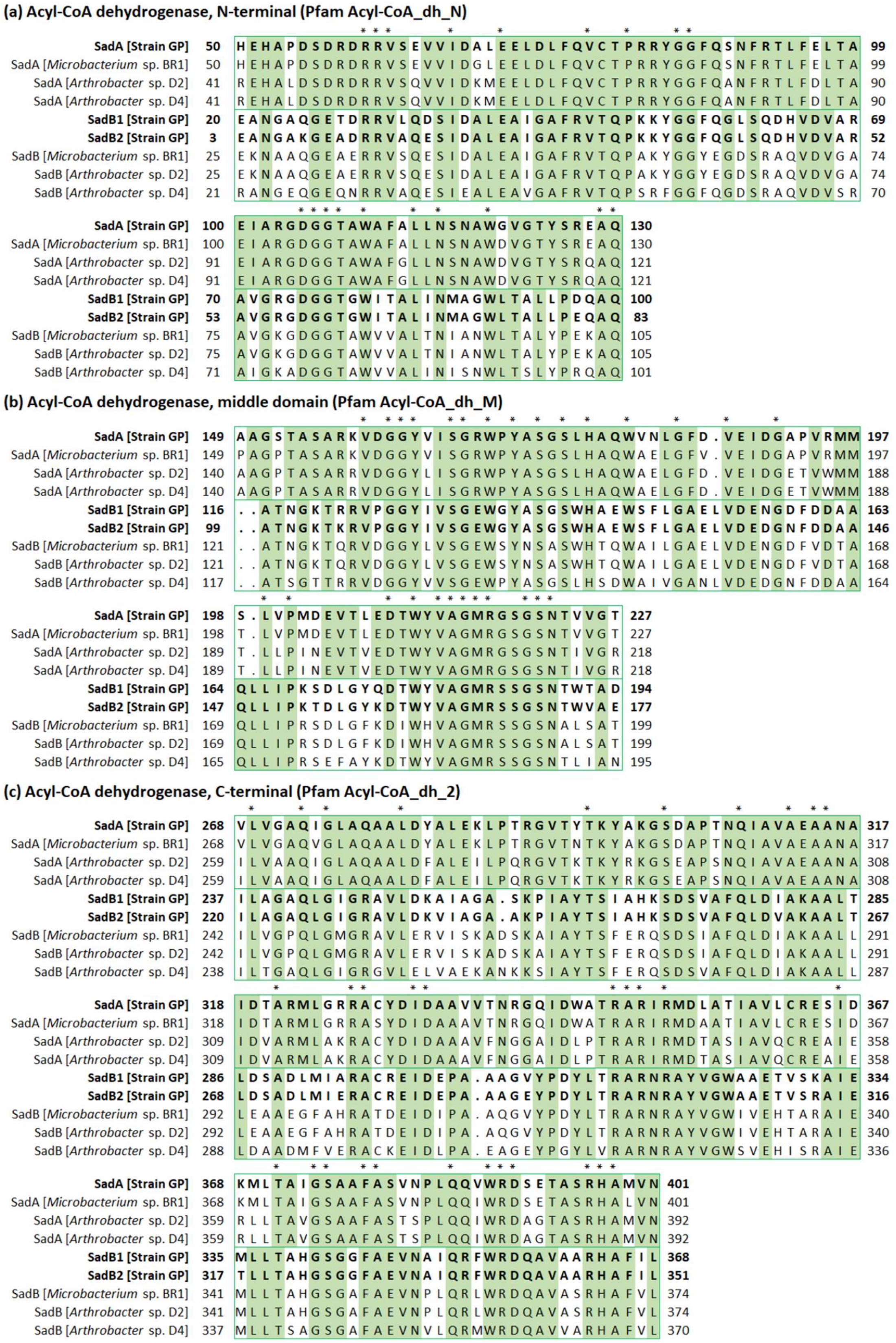

Supplementary Figure 8 –Amino acid alignment with MUSCLE [8] of Acyl-CoA domains: N-terminal (a), middle (b) and C-terminal (c); between SadA and SadB homologs in *Microbacterium* sp. BR1, *Arthrobacter* sp. D2 and D4 and strain GP (SadB1: D3X82_00235; SadB2: D3X82_03160). Conserved regions within SadA and SadB and highlighted in green and conserved regions shared between all proteins are marked with an asterisk.

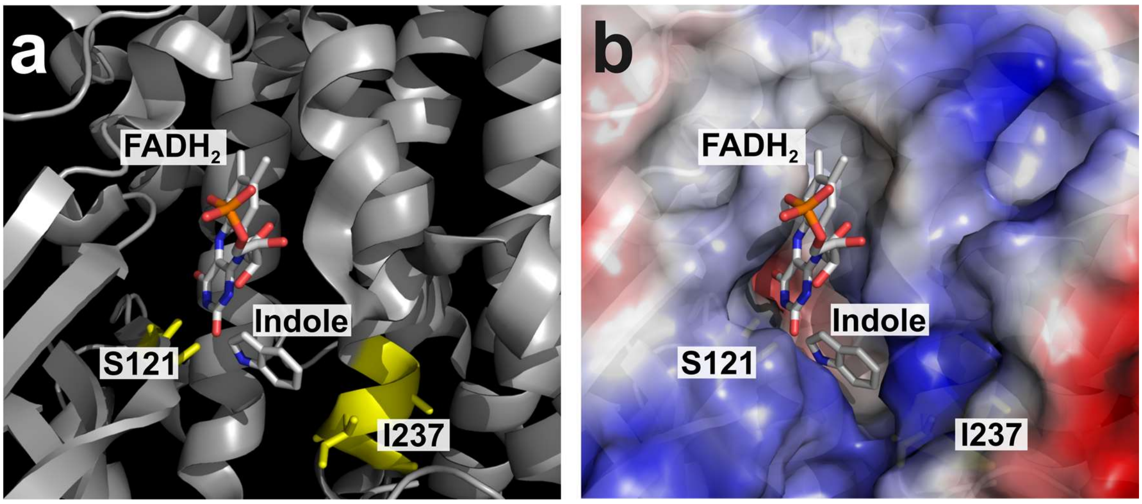

Supplementary Figure 9 – Close-up of the substrate-binding pocket of XiaF (PDB: 5LVW) bound to $FADH_2$ and indole obtained by Kugel et al. [9]. $FADH_2$ is the co-factor, indole the substrate and S121 and I237 are the residues that are modified in SadA of *Microbacterium* sp. BR1 and strain GP. The ribbon (a) and electrostatic surface potential (b) diagrams have been prepared with PyMol [10]. In b negative potential is shown in red and positive potential in blue.

References

1. Kyselková M, Kopecký J, Felföldi T, Čermák L, Omelka M, Grundmann GL, et al. Development of a 16S rRNA gene-based prototype microarray for the detection of selected actinomycetes genera. Antonie van Leeuwenhoek, Int J Gen Mol Microbiol. 2008;94:439–53.

2. Sanguin H, Remenant B, Dechesne A, Thioulouse J, Vogel TM, Nesme X, et al. Potential of a 16S rRNA-based taxonomic microarray for analyzing the rhizosphere effects of maize on *Agrobacterium* spp. and bacterial communities. Appl Environ Microbiol. 2006;72:4302–12.

3. Pernthaler J, Glöckner F-O, Schönhuber W, Amann R. Fluorescence in situ hybridization (FISH) with rRNA-targeted oligonucleotide probes. Methods Microbiol. 2001;30:207–26. doi:10.1016/s0580-9517(01)30046-6.

4. Tamura K, Stecher G, Peterson D, Filipski A, Kumar S. MEGA6: Molecular Evolutionary Genetics Analysis Version 6.0. Mol Biol Evol. 2013;30:2725–9. doi:10.1093/molbev/mst197.

5. Rambaut A. FigTree. http://tree.bio.ed.ac.uk/software/figtree/. Accessed 24 Jan 2019.

6. Contreras-Moreira B, Vinuesa P. GET_HOMOLOGUES, a versatile software package for scalable and robust microbial pangenome analysis. Appl Environ Microbiol. 2013;79:7696–701. doi:10.1128/aem.02411-13.

7. Thorvaldsdóttir H, Robinson JT, Mesirov JP. Integrative Genomics Viewer (IGV): High-performance genomics data visualization and exploration. Brief Bioinform. 2013;14:178–92.

8. Edgar RC. MUSCLE: a multiple sequence alignment method with reduced time and space complexity. BMC Bioinformatics. 2004;5:113. doi:10.1186/1471-2105-5-113.

9. Kugel S, Baunach M, Baer P, Ishida-Ito M, Sundaram S, Xu Z, et al. Cryptic indole hydroxylation by a non-canonical terpenoid cyclase parallels bacterial xenobiotic detoxification. Nat Commun. 2017;8:15804. doi:10.1038/ncomms15804.

10. Schrödinger L. The PyMOL molecular graphics system. 2002;Version 1.8.
